# Scavenger Receptor C Mediates Phagocytosis of White Spot Syndrome Virus and Restricts Virus Proliferation in Shrimp

**DOI:** 10.1371/journal.ppat.1006127

**Published:** 2016-12-27

**Authors:** Ming-Chong Yang, Xiu-Zhen Shi, Hui-Ting Yang, Jie-Jie Sun, Ling Xu, Xian-Wei Wang, Xiao-Fan Zhao, Jin-Xing Wang

**Affiliations:** Shandong Provincial Key Laboratory of Animal Cells and Developmental Biology, School of Life Sciences, Shandong University, Jinan, Shandong, China; University of Pennsylvania School of Medicine, UNITED STATES

## Abstract

Scavenger receptors are an important class of pattern recognition receptors that play several important roles in host defense against pathogens. The class C scavenger receptors (SRCs) have only been identified in a few invertebrates, and their role in the immune response against viruses is seldom studied. In this study, we firstly identified an SRC from kuruma shrimp, *Marsupenaeus japonicus*, designated *Mj*SRC, which was significantly upregulated after white spot syndrome virus (WSSV) challenge at the mRNA and protein levels in hemocytes. The quantity of WSSV increased in shrimp after knockdown of *Mj*SRC, compared with the controls. Furthermore, overexpression of *Mj*SRC led to enhanced WSSV elimination via phagocytosis by hemocytes. Pull-down and co-immunoprecipitation assays demonstrated the interaction between *Mj*SRC and the WSSV envelope protein. Electron microscopy observation indicated that the colloidal gold-labeled extracellular domain of *Mj*SRC was located on the outer surface of WSSV. *Mj*SRC formed a trimer and was internalized into the cytoplasm after WSSV challenge, and the internalization was strongly inhibited after knockdown of *Mj*β-arrestin2. Further studies found that *Mj*β-arrestin2 interacted with the intracellular domain of *Mj*SRC and induced the internalization of WSSV in a clathrin-dependent manner. WSSV were co-localized with lysosomes in hemocytes and the WSSV quantity in shrimp increased after injection of lysosome inhibitor, chloroquine. Collectively, this study demonstrated that *Mj*SRC recognized WSSV via its extracellular domain and invoked hemocyte phagocytosis to restrict WSSV systemic infection. This is the first study to report an SRC as a pattern recognition receptor promoting phagocytosis of a virus.

## Author Summary

Shrimp aquaculture is a major industry in many coastal countries, where it contributes significantly to socio-economic development. However, during the rapid expansion of shrimp industry, a succession of previously unknown diseases, especially viral diseases, have emerged in farmed shrimp and spread rapidly across international boundaries, which have significant effects on shrimp production. Since 1994, it has been estimated that annual losses globally caused by viral diseases have been as high as $US3 billion. White spot syndrome, caused by white spot syndrome virus (WSSV), is the most serious disease in shrimp aquaculture. Here, we reveal that a scavenger receptor in kuruma shrimp, *Marsupenaeus japonicus* (*Mj*SRC) functions as the pattern recognition receptor for WSSV. *Mj*SRC binds to WSSV and initiates phagocytosis of the virus, ultimately leading to degradation of WSSV in the lysosomes of hemocytes. This is the first report of the *Mj*SRC antiviral mechanism, which may be used as a control strategy to prevent WSSV infection in shrimp.

## Introduction

White spot syndrome virus (WSSV), which is a serious pathogen that threatens the aquaculture of shrimp, has led to huge economic losses in the shrimp industry [1]. Although great advances have been made in both pathogen study, including mechanisms and strategies used by the virus to infect and replicate in host cells, such as the findings of envelope proteins, VP19, 24, 26 and 28 interacting with each other and playing an important role in virus assembly and infection [2]; and host immune responses against the virus, including humoral and cellular immunity [3]. However, there is no existing treatment to restrict the uncontrolled occurrence and rapid spread of the disease in the field. Understanding the mechanisms of the host-virus interaction might help to find new strategies and methods for WSSV control.

Host innate immunity plays an important role in protecting the organism from pathogen invasion, particularly in invertebrates, which lack the typical adaptive immune responses [4]. Pattern recognition is the first step in the innate immune response, initiated by pattern recognition receptors (PRRs) that sense the presence of microorganic structural components, known as pathogen-associated molecular patterns (PAMPs) [2], such as lipopolysaccharides and peptidoglycan from bacteria or some proteins from viruses. Eleven types of PRRs, such as Toll like receptors, C-type lectins, thioester-containing protein and scavenger receptors, have been identified in shrimp [5]. Some receptors have been studied in depth; however, there are relatively few reports about the immunological roles of scavenger receptors (SRs) in invertebrates.

SRs comprise nine heterogeneous classes (A–I), classified in accordance with their multidomain structures [6]. Brown and Goldstein initially put forward the concept of SRs based on their ability to bind modified low-density lipoproteins (LDLs), such as acetylated or oxidized LDLs [7]. Now, SRs have been demonstrated to be members of PRRs, and play important roles in innate immunity [8]. The most studied SRs are SRA and B. SRA recognizes Gram-positive bacteria via binding to Lipoteichoic acid (LTA) [9]. Studies also showed that SRA could restrict hepatitis C virus replication by interacting with TLR3 in human hepatocytes [10]. CD36, which is the first cloned class B SR, is a sensor for LTA and diacylated lipopeptide, as well as a co-receptor for TLR2 in responses to microbial diacylglycerides [11].

SRC is a type 1 transmembrane glycoprotein comprising several distinct domains and is located on the external surface of the cytoplasmic membrane, with the C-terminal region in the cytoplasm. SRC has been identified in several invertebrates, such as *Drosophila melanogaster* and *Aedes aegypti*; however SRC has not been discovered in mammals. Previous studies showed that an SRC from *Drosophila* acted as a pattern recognition receptor to recognize and phagocytose both Gram-positive and Gram-negative bacteria [12]. However, the mechanism of viral phagocytosis is unclear, and whether SRC participates in viral phagocytosis remains largely unknown.

Clathrin-mediated endocytosis is probably the most common mechanism for endocytosis of small and medium size viruses [13–16]. Recent studies showed that WSSV entered hematopoietic tissue (HPT) cells or stomach epithelium via clathrin-mediated endocytosis or cholesterol (lipid raft) -dependent endocytosis mediated by C-type lectin-calreticulin interaction [17, 18]. Further studies indicated that WSSV could enter both hemocytes and HPT cells through endocytosis, but they could not replicate in hemocytes for some unknown reason [19]. Hemocytes are the major immune cells [19], especially in invertebrates. β-Arrestin1 and β-arrestin2 were originally discovered to internalize G protein-coupled receptors (GPCRs), such as the adrenergic receptor and μ-opioid receptor [20], into endosomes. β-Arrestins also participated in the internalization of many non-GPCR receptors or plasma membrane proteins, such as the type III transforming growth factor-β receptor and the insulin-like growth factor I receptor [21]. It was also identified as the adaptor protein for clathrin-mediated endocytosis [22, 23]. β-Arrestin1 and 2 were involved in the regulation of shrimp Toll pathway [24]. However, whether β-arrestins participate in the internalization of SRs has not been reported.

In this study, we obtained an SRC cDNA from the kuruma shrimp *M*. *japonicus*, and designated it as *Mj*SRC. Knockdown and overexpression analysis revealed that *Mj*SRC possessed antiviral function in shrimp. Further study found that *Mj*SRC appeared to oligomerize after WSSV challenge. We also discovered that hemocytes used clathrin-mediated endocytosis to engulf WSSV and that *Mj*β-arrestin2 was used as an adaptor protein to mediate the endocytotic process.

## Results

### *Mj*SRC is upregulated in shrimp challenged by WSSV

The open reading frame (ORF) region of *Mj*SRC encoded a protein of 692 amino acids, with a theoretical molecular mass of 73.84 kDa and an isoelectric point of 5.50 (GenBank accession number: KU213605). Two CCP (complement control protein) domains, one MAM (domain in meprin, A5, receptor protein tyrosine phosphatase mu) domain and one transmembrane region were identified in the protein. *Mj*SRC lacks the somatomedin B domain compared with insect SRCs (S1 Fig). The results of alignment and phylogenetic analysis of SRC from kuruma shrimp and other species showed that SRCs were not conserved, and that *Mj*SRC was relatively close to SRC from *Riptortus pedestris* in the phylogenetic tree (S2 Fig).

**Fig 1:**
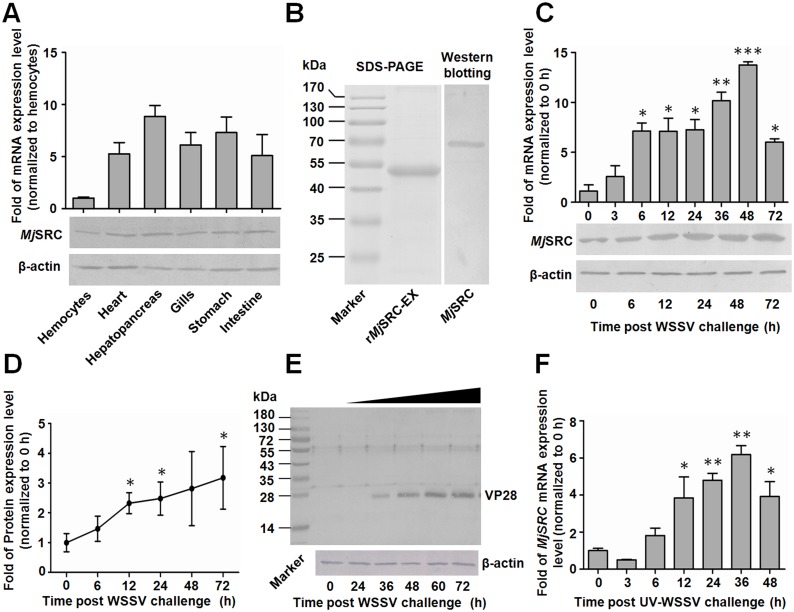
*Mj*SRC was upregulated in shrimp challenged by WSSV. (A) Tissue distribution of *Mj*SRC in shrimp. The mRNA expression level was analyzed using qRT-PCR (upper panel). The protein expression level was detected by western blotting (lower panel). β-Actin was used as the internal reference. (B) *Mj*SRC-EX recombinant expression in *E*. *coli*, analyzed by SDS-PAGE, and *Mj*SRC in hemocytes of shrimp, detected by western blotting using an anti-*Mj*SRC sera as the primary antibody. (C) Expression patterns of *Mj*SRC mRNA (upper panel) and protein (lower panel) in hemocytes of shrimp after WSSV challenge, detected by qRT-PCR and western blotting with the β-actin gene as the reference. Results were expressed as mean ± SD and analyzed statistically by Student’s *t*-test. *, *p* < 0.05, **, *p* < 0.01 and ***, *p* < 0.001. (D) The protein expression pattern of *Mj*SRC was digitalized using Quantity One software by scanning the western blotting bands from three independent repeats. Relative expression levels of *Mj*SRC/β-actin were expressed as the mean ± SD, and the value of the normal shrimp at 0 h was set as 1. Significant differences were analyzed by Student’s *t*-test. (E) VP28 expression levels analyzed by western blotting to detect WSSV replication in the gill. β-Actin was used as the sample loading control. (F) Expression patterns of *Mj*SRC mRNA in hemocytes of shrimp after UV-WSSV challenge, detected by qRT-PCR with the β-actin gene as the reference. Results were expressed as mean ± SD and analyzed statistically using Student’s *t*-test.

The distribution of *Mj*SRC transcripts was analyzed by quantitative real-time PCR (qRT-PCR), which revealed that *Mj*SRC was expressed in hemocytes, heart, hepatopancreas, gills, stomach and intestine (Fig 1A, upper panel). Anti-*Mj*SRC sera were prepared using the purified extracellular region, including CCP domains and MAM domain of *Mj*SRC (*Mj*SRC-EX) expressed in *Escherichia coli* (Fig 1B). Similarly, western blotting analysis showed that *Mj*SRC was distributed in hemocytes and other five tissues (Fig 1A, lower panel).

Temporal and spatial expression patterns of *Mj*SRC after WSSV challenge were analyzed using qRT-PCR and western blotting. The results of qRT-PCR showed that *Mj*SRC was significantly upregulated (from 7 to 13 folds) in hemocytes (Fig 1C, upper panel), but slightly increased in gills and intestine (S3A and S3B Fig), post WSSV injection. Western blotting analysis revealed that the protein expression levels of *Mj*SRC were also raised in hemocytes after WSSV challenge (Fig 1C lower panel and Fig 1D and S3C Fig). The VP28 protein was increased gradually from 36 to 72 h in gills after WSSV challenge (Fig 1E). Although VP28 was also increased in hemocytes after WSSV challenge, it was not much obvious comparing with that in gill (S3D Fig). Thus the VP28 level in gills was used to determine the viral replication in following studies. Ultraviolet inactivated WSSV (UV-WSSV) was also used to challenge shrimp. The mRNA expression levels of *Mj*SRC were detected by qRT-PCR and the results showed *Mj*SRC was upregulated significantly post UV-WSSV challenge (Fig 1F). Comparing with Fig 1C, the increasing folds (from 2 to 7 folds) of *Mj*SRC in UV-irradiated WSSV challenged shrimp was less than that in alive WSSV challenged shrimp. These results suggested that *Mj*SRC was involved in anti-WSSV infection, and the upregulation of *Mj*SRC was related with WSSV infection and replication in shrimp.

### *Mj*SRC restricts the replication of WSSV

**Fig 2:**
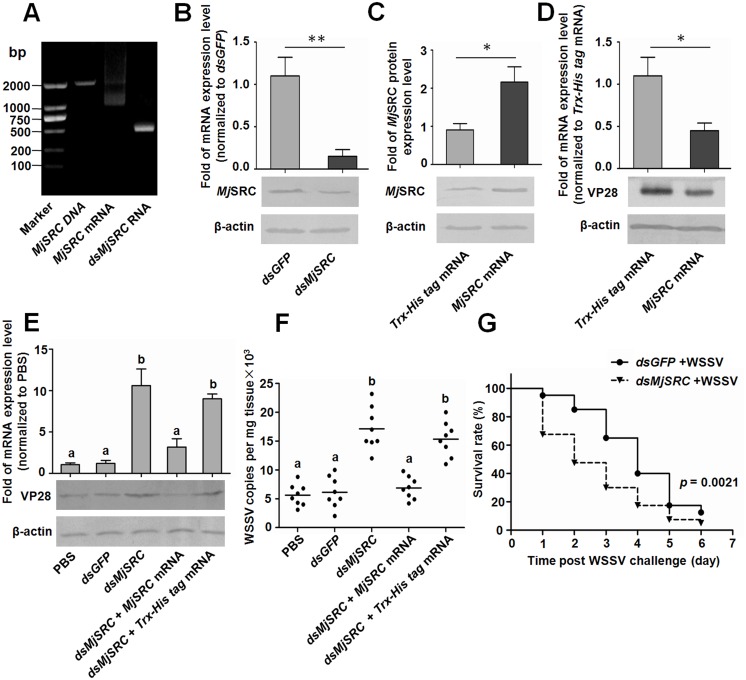
*Mj*SRC restricts WSSV replication in shrimp. (A) Agarose gel electrophoresis of in vitro amplified or synthesized cDNA, mRNA and dsRNA of *Mj*SRC, used in subsequent overexpression and RNAi assays. (B) Efficiency of *Mj*SRC RNAi in hemocytes, as detected by qRT-PCR (upper panel) and western blotting (lower panel). (C) Efficiency of *Mj*SRC overexpression in hemocytes, as detected by western blotting with anti-*Mj*SRC sera (lower panel). The protein expression level of *Mj*SRC was digitalized using Quantity One software by scanning the western blotting bands from three independent repeats (upper panel). (D) WSSV replication in shrimp after overexpression of *Mj*SRC. The shrimp was injected with WSSV after *MjSRC* mRNA injection. The VP28 expression in gills was determined at 48 h after WSSV injection using qRT-PCR (upper panel) and western blotting (lower panel). *Trx-His tag* mRNA overexpression was used as the control. (E) WSSV replication in *MjSRCi*-shrimp and *MjSRC*-rescue shrimp. Shrimp were divided into five groups, and WSSV replication was detected using qRT-PCR (upper panel) and western blotting (lower panel). Differences between each group were analyzed using one-way ANOVA. Different letters indicate statistical significance (*p* < 0.05). β-Actin was used as the internal reference. (F) The quantification of virion copies in gills from each individual shrimp in the five groups detected by qRT-PCR using the standard curve. Eight shrimp were used in each group. Differences between each group were analyzed using one-way ANOVA. Different letters indicate statistical significance (*p* < 0.05) and the same letter indicate no statistical difference (*p* > 0.05). (G) The survival rate of *Mj*SRC-RNAi shrimp infected with WSSV. Shrimp were divided into two groups (40 shrimp in each group). After 24 h of dsRNA injection, WSSV inoculums were injected. Shrimp survival was monitored every day after WSSV injection. *dsGFP* injection was used as the control. The survival rate of each group was calculated and the survival curves were presented as Kaplan-Meier plots. Differences between the two groups were analyzed with log-rank test using the software of GraphPad Prism 5.0. *p* = 0.0021.

To investigate the function of *Mj*SRC in WSSV infection of shrimp, RNA interference (RNAi) and overexpression of *Mj*SRC were conducted (Fig 2A) and WSSV replication in gills of *MjSRC* RNAi-shrimp and *MjSRC* overexpression-shrimp was detected via qRT-PCR and western blotting using VP28 as a marker. The mRNA and protein expression levels of *Mj*SRC were significantly reduced in hemocytes after *MjSRC* RNAi treatment (Fig 2B), and the effect of *Mj*SRC RNAi could last for 96 h (S4A Fig). The result of western blotting indicated that *Mj*SRC was overexpressed successfully in hemocytes of shrimp after injection of the *MjSRC* mRNA (Fig 2C and S4B Fig). WSSV was injected into shrimp after knockdown or overexpression of *MjSRC*. The results showed that the VP28 protein decreased markedly in the overexpression group at 48 h (Fig 2D) and 60 h (S4C Fig). Meanwhile, the VP28 expression in the *MjSRC*-RNAi group was distinctly higher than that in the *GFP*-RNAi group (Fig 2E). Furthermore, after rescuing the expression of *Mj*SRC in the RNAi group by *MjSRC* mRNA injection, the WSSV levels declined compared with the RNAi group (Fig 2E), indicating that the impaired antiviral effect in shrimp after RNAi of *Mj*SRC could be rescued by *Mj*SRC overexpression. To confirm the above results, the virus titers in gills of the five groups of shrimp were determined (Fig 2F), and the same results were obtained. Moreover, the survival rate of *MjSRC*-RNAi shrimp infected with WSSV decreased significantly compared with the control group (Fig 2G). All these results suggested that *Mj*SRC played an important role in antiviral immunity.

### *Mj*SRC functions in phagocytosis of WSSV in shrimp

**Fig 3:**
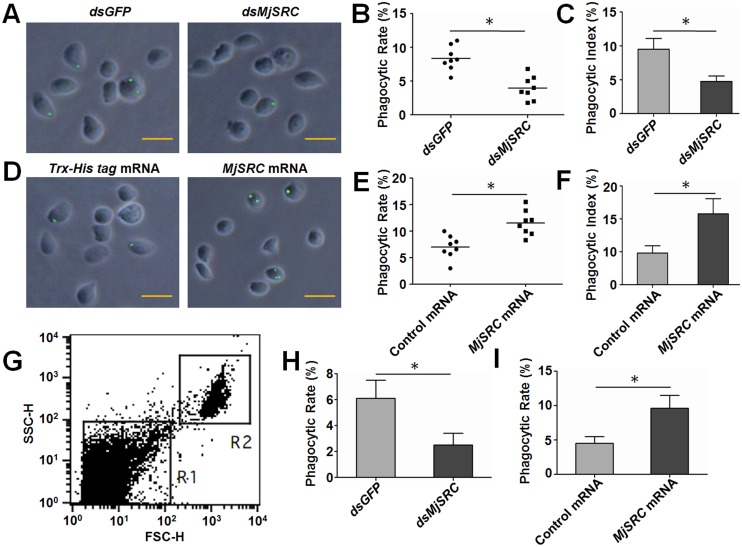
*Mj*SRC enhances the phagocytosis of WSSV in hemocytes of shrimp. (A-C) Phagocytic analysis after RNAi of *MjSRC*. (A) Hemocyte phagocytosis observed under the fluorescence microscope. WSSV virions were labeled with FITC (green) and then injected into shrimp. The hemocytes from three to five shrimp were collected 1 h after WSSV injection and stained with DAPI (blue) to label cell nuclei. Scale bar = 15 μm. (B) The phagocytic rate of hemocytes calculated by the formula in Materials and methods. Five hundred hemocytes were counted under the fluorescence microscope in each experiment. Injection of *dsGFP* was used as the control. Eight shrimp were used in each group. (C) The phagocytic index of hemocytes calculated by the formula in Materials and methods. (D-F) Phagocytic analysis after overexpression of *Mj*SRC. (D) Phagocytosis observed under the fluorescence microscope. (E) The phagocytic rate. (F) The phagocytic index. *Trx-His tag* mRNA was used as the control mRNA in the overexpression assays. (G) Phagocytosis was detected by flow cytometry. Intact hemocytes (R2), differentiated from virions and cell debris (R1), were analyzed only in this assay. (H and I) The phagocytic rate determined by flow cytometry after knockdown (H) or overexpression (I) of *Mj*SRC. Five thousand hemocytes were counted in each assay. The data were statistically analyzed using Student’s *t*-test. *, *p* < 0.05.

To investigate how *Mj*SRC reduced the amount of WSSV in shrimp, we analyzed the phagocytic rate after knockdown or overexpression of *Mj*SRC. The results of microscopic counting revealed that knockdown of *Mj*SRC decreased the phagocytic rate about 46% in hemocytes (Fig 3A and 3B), whereas the phagocytic rate was enhanced about 39% after *Mj*SRC overexpression (Fig 3D and 3E). Moreover, the phagocytic index of hemocytes after *Mj*SRC overexpression also increased significantly compared with the control group (Fig C and F). In addition, the change of hemocyte phagocytosis after knockdown or overexpression of *Mj*SRC could be observed visually in S6 Fig. Subsequently, flow cytometry was conducted to confirm the above results. Flow cytometry could differentiate hemocytes from virions and cell debris, and more than five thousand hemocytes in each group were analyzed (Fig 3G and S7 Fig). The results also showed that knockdown of *Mj*SRC reduced the phagocytotic rate (Fig 3H) and overexpression of *Mj*SRC promoted phagocytosis of WSSV in shrimp (Fig 3I). These results demonstrated that *Mj*SRC promoted phagocytosis of WSSV in shrimp.

### *Mj*SRC recognizes the envelope protein of WSSV

**Fig 4:**
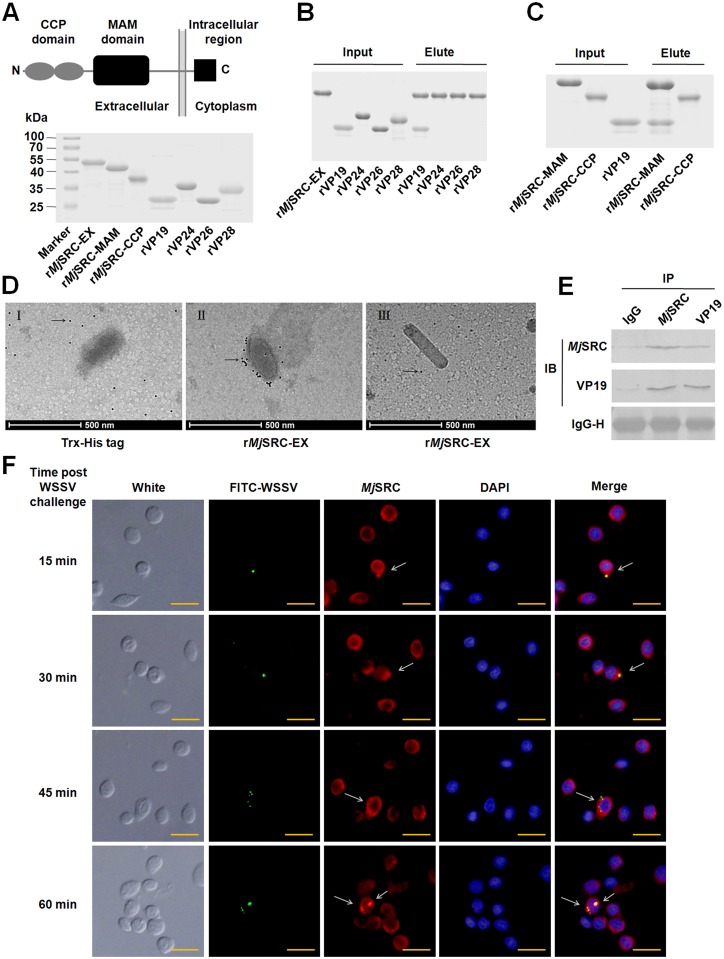
*Mj*SRC binds to the envelope protein of WSSV. (A) Schematic representation of *Mj*SRC indicating different domains (upper panel). Recombination and purification of GST-tagged r*Mj*SRC-EX, r*Mj*SRC-MAM, and r*Mj*SRC-CCP, as well as Trx-His-tagged rVP19, rVP24, rVP26 and rVP28 (lower panel). The proteins were analyzed using SDS-PAGE and stained with Coomassie blue. (B) GST-pulldown assays to detect the interaction between r*Mj*SRC-EX with rVP19, rVP24, rVP26 or rVP28. r*Mj*SRC-EX could bind to rVP19 only. (C) GST-pulldown assays to detect the interaction between different domains of *Mj*SRC, r*Mj*SRC-MAM and r*Mj*SRC-CCP, with rVP19. (D) The results of transmission electron microscopy. r*Mj*SRC-EX was labeled with colloidal gold (10 nm), and then incubated with purified WSSV virions on carbon-coated nickel grids. After thorough washing, the grids were observed under a transmission electron microscope. The Trx-His tag was also labeled with colloidal gold and then incubated with virions as the control (I). (II) Intact virion, (III) nucleocapsid of WSSV. The arrow indicated colloidal gold. Scale bar = 500 nm. (E) Co-IP assays to confirm the interaction between *Mj*SRC with VP19 *in vivo*. Anti-*Mj*SRC and anti-VP19 serum were used to analyze the interaction in hemocytes derived from WSSV-infected shrimp. Normal rabbit IgG was used as the negative control. (F) The co-localization of *Mj*SRC and FITC labeled-WSSV in shrimp hemocytes analyzed by immunocytochemistry. WSSV was labeled with FITC (green) and injected into shrimp. Hemocytes were collected at different time points (15, 30, 45 and 60 min) after WSSV injection. The primary antibody is anti-*Mj*SRC and the second antibody is anti-rabbit IgG Alexa-546 (red). Nuclei were stained with DAPI (blue). Scale bar = 15 μm.

To explore the mechanism of *Mj*SRC’s promotion of phagocytosis of WSSV, the interactions between *Mj*SRC and viral envelope proteins (VP19, VP24, VP26 and VP28) were investigated by pull-down assays *in vitro*. The recombinant proteins were expressed in *E*. *coli* (Fig 4A). The results of the GST-pull-down showed that the extracellular domains of *Mj*SRC (*Mj*SRC-EX) possessed binding activity with VP19, but not with the other three VP proteins of WSSV (Fig 4B). Further study indicated that the *Mj*SRC-MAM domain was responsible for this interaction (Fig 4C). Transmission electron microscopy showed that colloidal gold-labeled *Mj*SRC-EX could bind to the viral envelope but not the nucleocapsid of WSSV (Fig 4D); the Trx-His tag protein was used as the negative control. Co-immunoprecipitation (co-IP) was then performed to confirm the interaction between *Mj*SRC and VP19 *in vivo* (Fig 4E). In addition, the co-localization of *Mj*SRC and FITC labeled-WSSV in shrimp hemocytes was also detected at different time points, and the result showed that WSSV were attached to the cell surface at early time and co-localized with *Mj*SRC in cytoplasm of hemocytes 30 min post injection (Fig 4F). These results demonstrated that *Mj*SRC recognized WSSV via the direct interaction of its MAM domain with major envelope protein VP19 of WSSV *in vitro* and *in vivo*.

### *Mj*SRC oligomerizes to a trimer and is internalized from the membrane into the cytoplasm after WSSV infection

**Fig 5:**
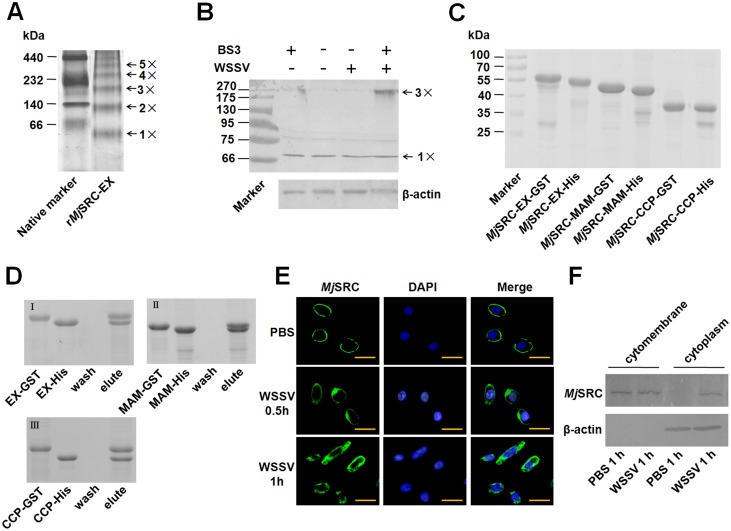
Oligomerization and internalization of *Mj*SRC in hemocytes of shrimp infected by WSSV. (A) Native PAGE of r*Mj*SRC-EX. Renatured r*Mj*SRC-EX (~ 60 KDa) and native protein marker (GE healthcare life science) were analyzed using native PAGE and stained with Coomassie blue. (B) A trimer of *Mj*SRC was detected *in vivo* using western blotting after treatment of hemocytes with crosslinker (BS3). Shrimp were divided into two groups: injection with PBS or WSSV, respectively. After 30 min, hemocytes were collected from each group and treated with BS3. These hemocytes were homogenized and separated by SDS-PAGE. Western blotting was then performed using anti-*Mj*SRC sera. β-Actin served as the reference. (C) Recombination and purification of GST-tagged r*Mj*SRC-EX, r*Mj*SRC-MAM, and r*Mj*SRC-CCP, as well as His-tagged r*Mj*SRC-EX, r*Mj*SRC-MAM, and r*Mj*SRC-CCP. These proteins were analyzed using SDS-PAGE and stained with Coomassie blue. (D) GST-pull-down assays to confirm the oligomerization of each domain of *Mj*SRC. (E) Immunocytochemistry was performed using anti-*Mj*SRC sera as the primary antibody. The secondary antibody was labeled with Alexa-488 (green). Cell nuclei were stained with DAPI (blue) and then observed under the fluorescence microscope. *Mj*SRC were located on the surface of hemocytes in normal shrimp (without WSSV challenge) (upper panel) and were then gradually internalized into cytoplasm at 0.5 h and 1 h of WSSV challenge (middle and lower panel). Scale bar = 15 μm. (F) Subcellular distribution of *Mj*SRC after WSSV challenge, as analyzed by western blotting. Proteins from the cytomembrane and cytoplasm of hemocytes were separated, respectively. Western blotting was performed using anti-*Mj*SRC sera. β-Actin served as a marker of the cytoplasm.

The oligomerization and subcellular location of *Mj*SRC were analyzed to observe its rapid response to WSSV infection. The oligomerization of r*Mj*SRC-EX was first analyzed using native PAGE, and the result showed that r*Mj*SRC-EX formed different oligomers *in vitro* (Fig 5A). Further studies showed that the native *Mj*SRC formed a trimer determined by molecular mass after WSSV challenge *in vivo* (Fig 5B). The amount of the trimer increased with the increase of crosslinker concentration (S8 Fig). Pull-down assays using different domains of *Mj*SRC indicated that both the MAM and CCP domains were involved in the formation of this oligomerization (Fig 5C and 5D). Immunofluorescence observation showed that *Mj*SRC was mainly localized in the cell membrane (Fig 5E upper panel). However, from 0.5 to 1 h of WSSV infection, *Mj*SRC was partially internalized into the cytoplasm (Fig 5E middle and lower panel). Proteins from the cytomembrane and cytoplasm of hemocytes were extracted and analyzed using western blotting. The result showed that *Mj*SRC could be detected only among the membrane proteins in control shrimp; however, it could be detected in the membrane and cytoplasm proteins in WSSV-infected shrimp (Fig 5F). These results suggested that WSSV challenge induced the oligomerization and internalization of *Mj*SRC in hemocytes.

### *Mj*β-arrestin2 interacts with *Mj*SRC and mediates *Mj*SRC internalization

**Fig 6:**
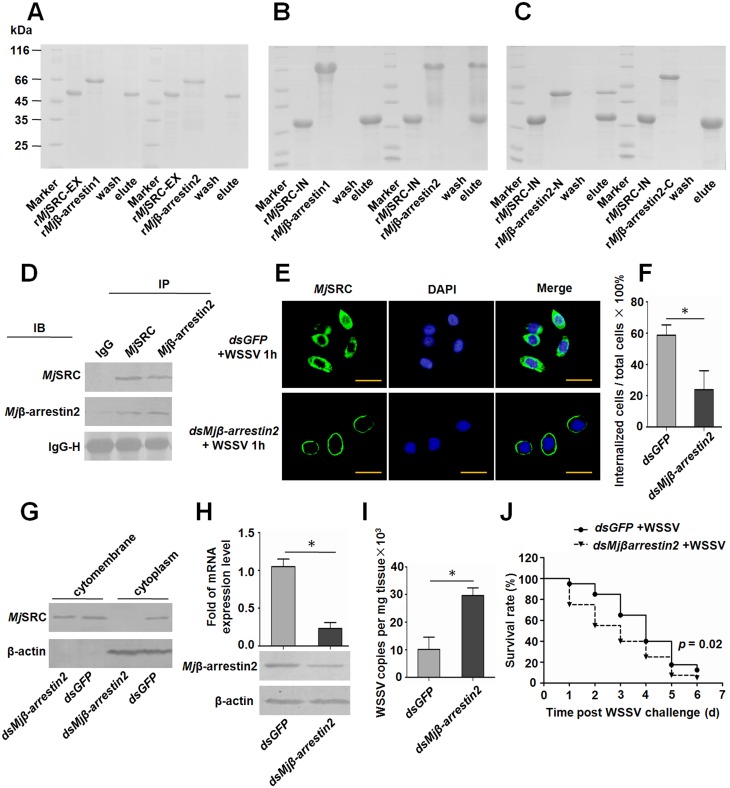
*Mj*β-arrestin2 interacts with *Mj*SRC and is involved in the internalization of *Mj*SRC in shrimp. (A) GST-pull-down to analyze the interaction of the *Mj*SRC extracellular region with *Mj*β-arrestin 1 and 2. (B) GST-pull-down to analyze the interaction of the *Mj*SRC intracellular region (*Mj*SEC-IN) with *Mj*β-arrestin 1 and 2. (C) GST-pull-down to analyze the interaction of *Mj*SEC-IN with the N- or C-terminal domain of *Mj*β-arrestin 2. (D) Co-IP was performed to detect the interaction between *Mj*SRC with *Mj*β-arrestin2 in shrimp using anti-*Mj*SRC and anti-*Mj*β-arrestin2 sera. (E) Shrimp were injected with *dsGFP* + WSSV, or *dsMjβ-arrestin2* + WSSV, respectively. After 1 h, hemocytes were collected from each group, and immunocytochemistry was performed using anti-*Mj*SRC sera as the primary antibody. The secondary antibody was labeled with Alexa-488 (green). Cell nuclei were stained with DAPI (blue) and then observed under the fluorescence microscope. Scale bar = 15 μm. (F) The percentage of cells showing *Mj*SRC internalization in total detected cells. Two hundred cells were counted under the fluorescence microscope in each group. The experiment and cell counting were performed three times. (G) Subcellular distribution of *Mj*SRC after knockdown of *Mj*β-arrestin2 in WSSV-infected shrimp, as analyzed by western blotting. β-Actin was used as a marker of cytoplasmic proteins. (H) Efficiency of *Mj*β-arrestin2 RNAi in hemocytes, as detected by qRT-PCR (upper panel) and western blotting (lower panel). (I) The quantification of viral copies from each group was detected by qRT-PCR. Significant differences were analyzed using Student’s *t*-test. *, *p* < 0.05. (J) The survival rate of *Mj*β-arrestin2-RNAi shrimp infected with WSSV. After 24 h of *dsRNA* injection, WSSV inoculums were injected. Dead shrimp was monitored every day after WSSV injection. *dsGFP* injection was used as the control. The survival rate was calculated and the survival curves were presented as Kaplan-Meier plots. Differences between the two groups were analyzed with log-rank test using the software of GraphPad Prism 5.0. *p* = 0.02.

To explore the mechanism of *Mj*SRC’s internalization, adaptor proteins were searched. We knew that β-arrestins participated in internalization of many non-GPCR receptors or plasma membrane proteins [21]. Two β-arrestins, *Mj*β-arrestin 1 and 2, were identified in shrimp (S9A and S9B Fig). Temporal and spatial expression of *Mj*β-arrestins in hemocytes were analyzed using qRT-PCR, which showed that *Mj*β-arrestin2 was upregulated significantly post WSSV challenge (S9C and S9D Fig). Pull-down assays were performed to analyze the interaction between *Mj*SRC and *Mj*β-arrestins, using the recombinant intracellular region of *Mj*SRC (*Mj*SRC-IN), *Mj*β-arrestin1, *Mj*β-arrestin2, and N-terminal and C-terminal domains of *Mj*β-arrestin2. The results showed that *Mj*β-arrestin2, rather than *Mj*β-arrestin1, interacted with *Mj*SRC-IN, and there was no interaction with *Mj*SRC-EX (Fig 6A and 6B). Further study showed that the N-terminal domain of *Mj*β-arrestin2 interacted with *Mj*SRC-IN (Fig 6C). The co-IP result also confirmed the interaction *in vivo* (Fig 6D). To analyze the interaction related with *Mj*SRC internalization, *Mjβ-arrestin2*-RNAi was performed and the subcellular localization of *Mj*SRC in hemocytes was detected (Fig 6E). The proportion of hemocytes that appeared to internalize *Mj*SRC decreased significantly after *dsMjβ-arrestin2* RNA injection compared with *dsGFP* RNA injection (Fig 6F). Western blotting analysis further confirmed the result (Fig 6G). To investigate whether the interaction of *Mj*SRC with *Mj*β-arrestin2 was related to the antiviral immune response, RNAi of *Mj*β-arrestin2 was conducted and the proliferation of WSSV and the survival rate were analyzed. The results showed that after knockdown of *Mjβ-arrestin2* (Fig 6H), virus titers increased significantly compared with the control shrimp (Fig 6I), and the survival rate of shrimp challenged by WSSV decreased in the *Mjβ-arrestin2*-RNAi group compared with the control group (Fig 6J). These results suggested that *Mj*β-arrestin2 participated in the internalization of *Mj*SRC by interaction with the intracellular region of *Mj*SRC. *Mj*β-arrestin2 also played an important role in antiviral immunity.

### *Mj*SRC-arrestin2-mediated hemocyte phagocytosis of WSSV is clathrin-dependent

**Fig 7:**
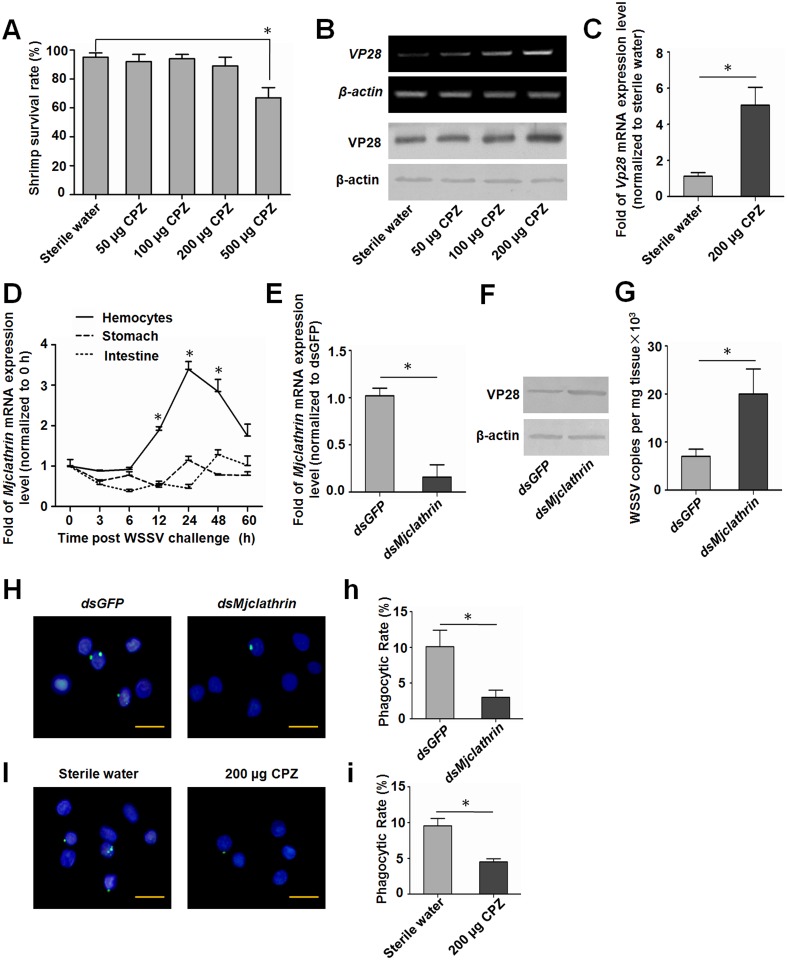
Hemocytes phagocytosis of WSSV is clathrin-dependent. (A) Effect of chlorpromazine (CPZ) on the viability of shrimp. Shrimp were treated with increasing concentrations of CPZ for 2 days and the survival rate was calculated. (B) Shrimp were challenged with WSSV, and divided into four groups. After 24 h of WSSV challenge, different concentrations of CPZ were injected into each group, respectively. Another 24 h later, *VP28* mRNA expression levels in the gills from each group were detected using RT-PCR (upper two panels) and the protein levels were analyzed using western blotting (lower two panels). (C) The amounts of WSSV DNA were compared between the two groups, using VP28 as a marker, and detected by qRT-PCR. The *β-Actin* gene served as the reference. (D) Expression patterns of *Mj*clathrin mRNA in hemocytes, stomach and intestine of shrimp after WSSV challenge, as detected by qRT-PCR. The *β-Actin* gene was used as the reference. Results were expressed as mean ± SD and analyzed statistically using Student’s *t*-test. *, *p* < 0.05. (E) Efficiency of *Mj*clathrin RNAi in hemocytes, as detected by qRT-PCR. (F) VP28 expression in *Mj*clathrin-RNAi shrimp were analyzed by western blotting. β-Actin served as the internal control. (G) The quantification of viral copies from each group was detected by qRT-PCR based on a standard curve. Shrimp were challenged with WSSV, 24 h later, *dsGFP* or *dsMjclathrin* RNA were injected into each group. The VP28 expression level or the viral copies were analyzed after 24 h of *dsRNA* injection. (H) Phagocytosis of hemocytes in *dsMjclathrin* knockdown shrimp observed under the fluorescence microscope. Shrimp were injected with *dsGFP* or *dsMjclathrin*. After 24 h, WSSV virions (labeled with FITC, green) were injected into each group. The hemocytes were collected 1 h after WSSV injection for observation. (h) Phagocytic rate was calculated based on the formula described in method. (I) The effect of inhibitor CPZ on phagocytic rate of hemocytes. After 1 h of CPZ injection, FITC-labeled WSSV were injected and 1 h later, hemocytes were collected. Cell nuclei were stained with DAPI (blue). (i) Phagocytic rate was calculated based on the formula described in method. Scale bar = 15 μm. Five hundred hemocytes were counted under the fluorescence microscope in each experiment. Student’s *t* test was used for statistic analysis. *, *p* < 0.05.

To explore whether the *MjSRC*-arrestin2-mediated phagocytosis of WSSV was clathrin-dependent, a chemical agent, chlorpromazine (CPZ), an effective inhibitor of clathrin-mediated endocytosis, was used to inhibit clathrin-mediated phagocytosis in shrimp. The toxic effect of CPZ in shrimp was firstly determined by calculating the survival rate of shrimp after CPZ injection. The results showed that low doses of CPZ (< 200 μg per shrimp) did not reduce the viability of shrimp (Fig 7A). After injection of CPZ into the WSSV-infected shrimp, VP28 expression at the mRNA, protein and genome level increased obviously compared with the control shrimp (Fig 7B and 7C), suggesting that hemocytes utilized clathrin-mediated endocytosis to engulf the invading WSSV. A clathrin heavy chain from kuruma shrimp was firstly identified and designated *Mj*clathrin (GenBank accession number: KU984437) (S10 Fig). *Mj*clathrin was widely distributed in several tissues of shrimp, and its mRNA levels were upregulated significantly after WSSV challenge in the hemocytes (Fig 7D), which suggesting *Mj*clathrin was involved in WSSV infection in shrimp. After knockdown of the expression of *Mj*clathrin in WSSV-infected shrimp (Fig 7E), the VP28 protein level increased compared with the control shrimp (Fig 7F), and the virus titers also increased significantly in *Mjclathrin*-RNAi shrimp (Fig 7G). Further analysis found that phagocytic rate of hemocytes decreased significantly after inhibition of *Mj*clathrin by *dsMjclathrin* RNA injection or CPZ injection (Fig 7H and 7I), which indicated that the phagocytosis of hemocytes mediated by *Mj*clathrin played an important role in restricting WSSV infection and replication in shrimp. All these results demonstrated that hemocytes took advantage of clathrin-mediated phagocytosis.

### Lysosomes were related to the clearance of WSSV in shrimp

**Fig 8:**
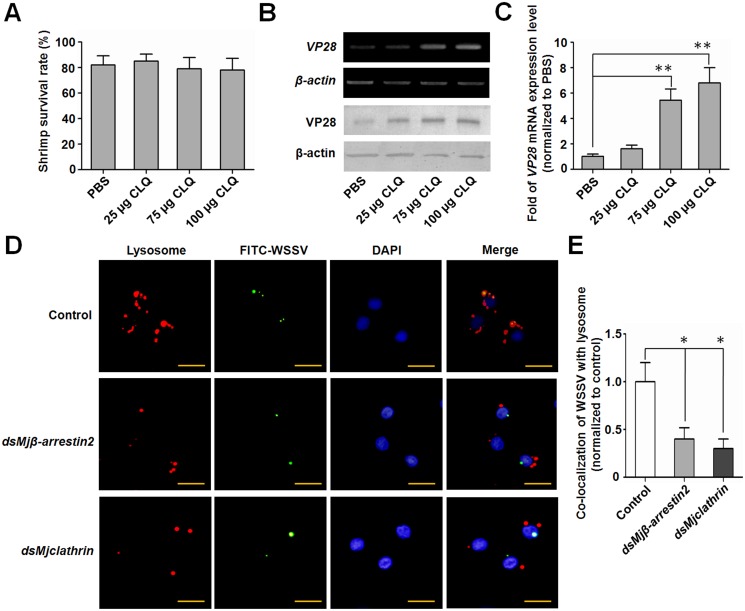
Lysosomes of hemocytes were involved in the clearance of WSSV in shrimp. (A) Effect of chloroquine (CLQ) on the viability of shrimp. Shrimp were treated with increasing concentrations of CLQ for 2 days and the survival rate was calculated. (B) Shrimp were divided into four groups, 24 h post WSSV challenge, different concentrations of CLQ were injected into each group, respectively. Another 24 h later, *VP28* mRNA expression levels in the gills from each group were detected using RT-PCR (upper two panels) and the protein levels were analyzed using western blotting (lower two panels). (C) *VP28* mRNA expression levels in the gills from each group were detected by qRT-PCR. Results are expressed as mean ± SD and were analyzed statistically using Student’s *t*-test. **, *p* < 0.01. (D) Co-localization of ingested virions and lysosomes. After 24 h of *dsMjβ-arrestin2* or *dsMjclathrin* injection, FITC-labeled WSSV virions were injected into shrimp. After another 1 h, hemocytes were collected, incubated with LysoBrite Red (BBI) to label lysosomes, stained with DAPI and then observed under a fluorescence microscope. Scale bar = 15 μm. *dsGFP* injection was used as the control. (E) Statistical analysis of the co-localization between WSSV and lysosomes. The amounts of co-localized WSSV with lysosomes were counted under the fluorescence microscope in each group. The rate of co-localization of WSSV and lysosome in ds*Mj*β-arrestin2- or ds*Mj*clathrin-RNAi group was normalized to the control group. Student’s *t*-test was used for statistic analysis. *, *p* < 0.05.

To invesgate whether lysosomes was involved in inhibition of WSSV, the colocalization of WSSV and Lysosome was analyzed. Immunocytochemical analysis was performed using hemocytes collected from shrimp injected with FITC-labeled WSSV. The result showed that the labeled WSSV virions were co-localized with lysosomes which stained with LysoBrite Red in hemocytes (Fig 8D, upper panel). After knockdown of *Mj*β-arrestin2 or *Mj*clathrin, the co-localization of WSSV with lysosomes was all decreased significantly in hemocytes (Fig 8D, lower two panels and Fig 8E). To confirm lysosomes were involved in WSSV degradation, the lysosome inhibitor chloroquine (CLQ) was injected into shrimp, and WSSV proliferation was detected. Low doses (<100 μg per shrimp) of CLQ did not reduce the viability of shrimp (Fig 8A). After injection of CLQ into the WSSV-infected shrimp, VP28 expression at the mRNA and protein levels increased obviously compared with the control shrimp (Fig 8B and 8C), suggesting that lysosomes were involved in elimination of WSSV. All these results together showed *Mj*β-arrestin2-*Mj*clathrin-lysosomes pathway promoted degradation of WSSV in shrimp.

## Discussion

In this study, we firstly identified a class C scavenger receptor (*Mj*SRC) from *M*. *japonicus*, which worked as a phagocytotic receptor of WSSV on shrimp hemocytes. *Mj*SRC interacts with the VP19 WSSV envelope protein and *Mj*β-arrestin2 to promote hemocyte phagocytosis in a clathrin-dependent manner and inhibits WSSV replication and propagation in shrimp.

Scavenger receptors (SRs) represent a superfamily of structurally unrelated, distinct gene products, which are subdivided into different classes, mostly by their shared functional properties [6]. Eight members of SRs in mammals, including SRA-SRI, but not SRC, which only exists in invertebrates, have been reported in recent years, and their properties have been gradually discovered [25–29]. SRs function to recognize modified self molecules (such as Ox-LDL and Ac-LDL) and several non-self components, e.g. bacterial LPS and LTA, and contribute to a range of physiological or pathological processes, including promotion of the internalization of their ligands and activation of downstream signaling pathways [6, 8]. *Mj*Croquemort and *Mj*SR-B1, the only two SR family members identified and characterized in shrimp to date, play a specific role in the phagocytosis of bacteria [30, 31]. SRC proteins have been identified in several species, such as *Anopheles darlingi*, *Aedes aegypti*, *Bombyx mori* and *D*. *melanogaster* [32–34]. However, there have been few reports concerning their biological roles. Four isoforms of SRC in *D*. *melanogaster* have been identified, one of which (dSR-CI) mediates phagocytosis of Gram-positive and Gram-negative bacteria [12]. A Recent study showed that an SRC from *A*. *aegypti* (AaSRC) could recognize dengue virus and regulate the expression of antimicrobial peptides to eliminate the virus [35]. In this study, we identified an SRC from kuruma shrimp (*Mj*SRC), which contained two CCP domains, a MAM domain, a transmembrane region and an intracellular region (S1 Fig). *Mj*SRC played an effectively antiviral role by enhancing hemocytes phagocytosis of WSSV in shrimp.

Several phagocytic receptors have been shown to be involved in bacterial recognition and uptake. There are several microbial ligands for phagocytic receptors, include various proteins, and complex lipids, such as lipopolysaccharides (LPS), lipoteichoic acids, and mycobacterial lipids [36]. For example, SR-A mediates non-opsonic phagocytosis of several bacterial pathogens, including *Neisseria meningitides* [37], by recognizing LPS. dSR-CI is a macrophage-specific PRR that recognizes Gram-positive and Gram-negative bacteria by binding to a broad range of polyanionic ligands. The CCP domains, together with the MAM domain, of dSR-CI are sufficient to bind bacteria *in vitro* [12]. *Mj*SR-B1 serves as a phagocytosis receptor, recognizing Gram-positive and Gram-negative bacteria by binding different polysaccharides in kuruma shrimp [31]. In this study, we identified that *Mj*SRC is the receptor of virus WSSV. The colloidal gold-labeled *Mj*SRC were coated on the outer surface by WSSV under transmission electron microscope (Fig 4), indicating that *Mj*SRC bound to WSSV directly. Pull-down assays showed that the MAM domain of the receptor interacted with VP19. Co-IP assays confirmed the interaction between *Mj*SRC with VP19 *in vivo*. The co-localization detection of *Mj*SRC and FITC labeled-WSSV in shrimp hemocytes was also confirmed the result (Fig 4F). VP19, VP24, VP26 and VP28 are the four major envelope proteins of WSSV and function in virus entry and systemic infection [38–40]. VP24, VP26 and VP28 share high sequence homology with each other; however, VP19 has a unique structure and biological character [41, 42]. The CCP domain exists mainly in complement system proteins and promotes the formation of oligomers [43]. MAM is an extracellular domain and exists in many kinds of proteins, such as meprin, neuropilins and zonadhesins [44]. In addition, MAM domain mediates protein-protein interactions and is involved in homo-oligomerization for neuropilin-1/-2 and meprin A [45, 46]. In this study, we found that after WSSV infection, *Mj*SRC was upregulated and oligomerized to a trimer that recognized WSSV via binding to VP19 to initiate antiviral responses in shrimp. We also discovered that UV-inactivated WSSV could upregulated *Mj*SRC expression level in hemocytes, but the increasing folds were lower compared to that the virus was not irradiated for shrimp challenge (Fig 1F). This suggested that the upregulation of *Mj*SRC in hemocytes was related with WSSV infection and replication in shrimp.

Uptake of a microbial particle usually occurs via phagocytosis induced by the pathogen-receptor interaction. Phagocytosis, defined as the cellular uptake of particles within a plasma-membrane envelope, is closely related to the endocytosis of ligands by macropinocytic and receptor pathways [47]. Viruses can enter host cells by endocytosis, macropinocytosis or fusion [48]. SRs are involved in phagocytosis of microbes, but the exact mechanisms remain unclear. In our study, we found that *Mj*SRC could form homo-trimers via its CCP and MAM domains after WSSV challenge (Fig 5), just like a number of other PRRs, which form oligomers after recognizing pathogens, such as SRA, NOD-like receptor, Toll receptor, acetylcholine receptor and GPCR [49–52]. Moreover, *Mj*SRC was subsequently internalized from the plasma membrane into the cytoplasm after WSSV challenge (Fig 5). As essential scaffolding proteins, β-arrestins are involved in multiple signaling pathways activated in host cells by pathogens, participating in innate immunity and the inflammatory response [53]. β-Arrestins regulate G-protein-coupled receptor (GPCR) signaling as G-protein-independent signal transducers [54]. They can scaffold various components of the ERK cascade, bringing them into close proximity to promote ERK activation [55]. β-arrestins were found to promote clathrin-mediated internalization of GPCRs by interacting with, and scaffolding various components of, the clathrin-mediated endocytosis machinery. In particular, β-arrestin2 promotes the activation of ARF6 and endocytosis in the migration of vascular smooth muscle cells [56]. After overexpression in mouse embryonic fibroblasts, β-arrestin2 also enhanced low density lipoprotein receptor (LDLR) endocytosis (by 65%), through the interaction between β-arrestin2 with LDLR cytoplasmic tail [57]. The C-terminus of β-arrestin2 is reported as the binding domain of clathrin [58, 59]. In this study, we found that *Mj*β-arrestin2 was upregulated by WSSV challenge, and the intracellular region of *Mj*SRC (*Mj*SRC-IN) interacted with N-terminus of *Mj*β-arrestin2. *Mj*SRC internalization decreased significantly after RNAi of *Mjβ-arrestin2*, whilst the amount of WSSV increased significantly (Fig 6). Our study suggested that β-arrestin2 was an adaptor protein for the *Mj*SRC receptor and was responsible for receptor internalization.

Virus endocytic entry occurs in a stepwise manner, including attachment to the cell surface, clustering of receptors, activation of signaling pathways, formation of endocytic vesicles and vacuoles, delivery of viral cargo to endosomal compartments and escape into the cytosol [60]. Clathrin-coated vesicles are responsible for an important form of endocytosis for many kinds of virions [60], including WSSV in crayfish [17]. Usually this kind of endocytosis is beneficial for virus infection. Studies showed that WSSV virions could enter hemocytes; however, their genome replication and structural protein expression were unsuccessful in crayfish hemocytes [19]. The VP28 protein expression level was upregulated in the gills after WSSV challenge (Fig 1E), hematopoietic tissue cells [19] and stomach [18], but not in hemocytes after WSSV challenge in shrimp. All the results suggested that hemocytes had a mechanism to restrict WSSV replication. A recent study showed that hemocytes’ phagocytosis contributed to the virus-specific immune response in *D*. *melanogaster*, especially to cricket paralysis virus, Flock House Virus and vesicular stomatitis virus [61]. We discovered that *Mj*clathrin was upregulated in hemocytes after WSSV challenge. The viral titer increased in *Mjclathrin*-RNAi shrimp that were infected with WSSV 24 h before *dsRNA* injection. CPZ injection also resulted in upregulation of the VP28 expression level. Further study showed that phagocytosis of WSSV by hemocytes was inhibited after *dsMjclathrin* or CPZ injection (Fig 7). Therefore, the *Mj*SRC-arrestin-clathrin pathway is involved in the phagocytosis of WSSV to restrict viral infection in shrimp. After internalization of viruses, phagosomes subsequently fuse with intracellular granules to form the phagolysosome, within which microbial killing is achieved by a combination of non-oxidative and oxidative mechanisms [48, 62]. In this study, the co-localization of ingested virions and lysosomes was observed in hemocytes (Fig 8). In addition, the amount of WSSV increased significantly in shrimp after inhibition of lysosome by CLQ compared with control, indicating that lysosome in hemocytes is probably involved in the clearance of WSSV.

In our study, we found that there were 50–60% hemocytes showing *Mj*SRC internalization after WSSV challenge (Fig 6F). However, the phagocytic rate was lower than internalization rate in the shrimp. We think that there are several reasons for the differences: Usually virions could not be observed under the microcope and only the block of the virions could be observed under the microscope. The WSSV is an envelope virus and the envenlope can be easily destroyed in the purification. Therefore the injected FITC labeled WSSV contains some debris with envelope proteins and these proteins can interact with *Mj*SRC and induce its internalization, but the debris could not be observed in hemocytes under the microscope. Another reason is the FITC is easily quenching under the fluorescence microscope. Therefore, phagocytic rate of hemocytes is lower than that of internalization.

**Fig 9:**
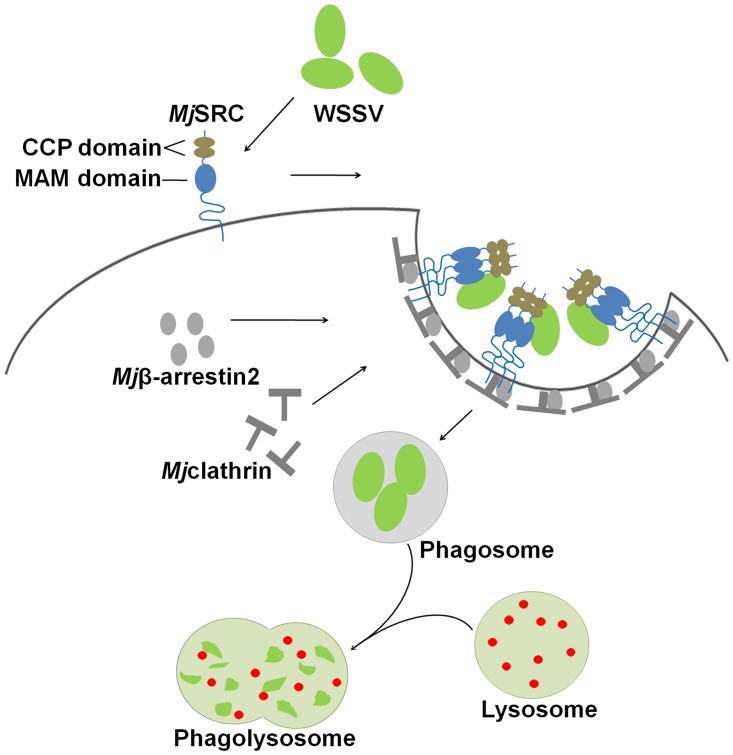
Schematic representation of the antiviral mechanism of *Mj*SRC. *Mj*SRC locates on the cell membrane of hemocytes, with two CCP domains and one MAM domain projected out of the cell. After WSSV infection, *Mj*SRC oligomerized to a trimer via its CCP and MAM domains. Subsequently, the intracellular region of *Mj*SRC recruits and interacts with an adaptor protein, *Mj*β-arrestin2, and the latter interacts with *Mj*clathrin to induce internalization of *Mj*SRC with WSSV via a clathrin-coated vesicle. This vesicle that holds the foreign pathogens (WSSV) is called a phagosome, which becomes a phagolysosome after fusing with a lysosome. Ultimately, WSSV is eliminated by various enzymes.

In conclusion, *Mj*SRC was identified to serve as a WSSV phagocytotic receptor in *M*. *japonicus*. *Mj*SRC recognizes WSSV by the interaction of *Mj*SRC-MAM with VP19. After *Mj*SRC oligomerization, the intracellular domain of *Mj*SRC recruits and binds to *Mj*β-arrestin2, which initiates the phagocytosis of WSSV by hemocytes in a clathrin-dependent manner. WSSV are finally degraded in hemocyte phagolysosomes, which effectively restricts viral infection in shrimp (Fig 9). This is the first report of an *Mj*SRC-mediated antiviral mechanism in shrimp.

## Materials and Methods

### Animals

Healthy kuruma shrimp, *M*. *japonicus*, about 8–10 g each, were obtained from an aquatic product market in Jinan, Shandong province, China. Shrimp were cultured in aerated artificial seawater with a salinity of about 22% (w/v), and fed with a commercial diet daily. Animals were kept for 2 days at 22°C before use and were selected randomly in each experiment.

### Preparation of WSSV inoculums and challenge of shrimp

The WSSV inoculum was acquired according to previous publications [18, 63]. Briefly, all tissues (except the hepatopancreas) of WSSV-infected shrimp were homogenized in sterile PBS (137 mM NaCl, 2.7 mM KCl, 2mM KH2PO4, 10 mM Na2HPO4, pH 7.4) at a ratio of 10% (w/v). After centrifugation at 3000 × g for 5 min at 4°C, the supernatant was filtered through a 0.45-nm filter. The virus titer was determined by quantitative real-time PCR, on the basis of a previous report [18]. Each shrimp was injected a 30 μL of WSSV inoculum (1×10^5^ virions) in the subsequent challenge experiments. Ultraviolet inactivated WSSV (UV-WSSV) was also used to challenge shrimp. WSSV inoculums were irradiated with ultraviolet for 20 min and then 30 μL inoculums (about 1×10^5^ virions) were injected into each shrimp.

### Quantification of WSSV copies

The quantification of WSSV copy numbers was performed using a previously reported method [63, 64]. Briefly, a WSSV VP28 fragment was amplified and inserted into the plasmid (pBlueScript II SK+). The recombinant plasmid was quantified using a spectrophotometer (GeneQuant, Amersham Biosciences, Piscataway, NJ, USA). Using the known molecular weight of the plasmid, its copy number could be calculated. Subsequently, the plasmid was diluted gradually (10^9^, 10^8^, 10^7^, 10^6^, 10^5^, 10^4^), and the diluted samples were used as templates for qRT-PCR with primers VP28-RT-F and VP28-RT-R (Table 1). The cycle threshold (CT) and the quantity of the template were used to make a standard curve for WSSV quantification (S5 Fig). The genomic DNA extracted from the viral inoculum or WSSV-infected tissue, together with the gradient diluted plasmid samples, were analyzed by qRT-PCR to obtain the absolute copies of WSSV from the inoculums or infected tissue.

**Table 1:** Primers used in this study.

Primers	Sequence (5′-3′)
**Real-time PCR**	
*Mj*SRC-RT-F	TCTCCCACAGAGGCTACTTC
*Mj*SRC-RT-R	CGCTTCGGTCGTTTGATT
*Mj*β-arrestin2-RT-F	TGGCTCTATTCCTCTGCG
*Mj*β-arrestin2-RT-R	ATCTGCCCCGAACATGC
VP28-RT-F	CTCCGCAATGGAAAGTCTGA
VP28-RT-R	GGGTGAAGGAGGAGGTGTT
*Mj*clathrin-RT-F	ATTTGATAGAGTTTCGTCG
*Mj*clathrin-RT-R	GCAGGTCGTAGCAGTGGA
β-actin-RT-F	GCATCATTCTCCATGTCGTCCCAGT
β-actin-RT-R	TACGGCTGCGAGAAGACGACAGAA
**Protein expression**	
*Mj*SRC-ORF-F	TACTCAGAATTCATGGCTACTAAGTATTCG
*Mj*SRC-ORF-R	TACTCAGCGGCCGCTCAGGCCACCGTGCTCCG
*Mj*SRC-EX-F	TACTCAGAATTCTGCCCGAAGATCCTGCGT
*Mj*SRC-EX-R	TACTCAGCGGCCGCGTGGAAGCACTCGTCCCC
*Mj*SRC-IN-F	TACTCAGAATTCTGGAGAGTCCGCCGCCAG
*Mj*SRC-IN-R	TACTCAGCGGCCGCGGCCACCGTGCTCCGGGG
*Mj*SRC-CCP-F	TACTCAGAATTCTGCCCGAAGATCCTGCGT
*Mj*SRC-CCP-R	TACTCAGCGGCCGCACACACGGGCGCCGTGTC
*Mj*SRC-MAM-F	TACTCAGAATTCGCGGAGGAGTTATCGTGC
*Mj*SRC-MAM-R	TACTCAGCGGCCGCGTGGAAGCACTCGTCCCC
VP19-F	CGCGGATCCATGGCCACCACGACTAACAC
VP19-R	CCGCTCGAGTTAATCCCTGGTCCTGTTCTTAT
VP24-F	TACTCAGAATTCAACATAGAACTTAACAAGAAAT
VP24-R	TACTCACTCGAGGCCAGGAGAAAAACGCAT
VP26-F	TACTCAGAATTCACACGTGTTGGAAGAAGCGT
VP26-R	TACTCACTCGAGCTTCTTCTTGATTTCGTCCTTG
VP28-F	TACTCAGAATTCATGGATCTTTCTTTCACTCT
VP28-R	TACTCACTCGAGTTACTCGGTCTCAGTGCCAG
*Mj*β-arrestin2-ORF-F	TACTCAGAATTCGTCAGTATGCCTACAAATCTA
*Mj*β-arrestin2-ORF-R	TACTCACTCGAGTCTCTAATATGGCTGTAC
*Mj*β-arrestin2-C-F	TACTCAGAATTCATCCAGTGCAATGAGCAT
*Mj*β-arrestin2-C-R	TACTCACTCGAGTCTCTAATATGGCTGTAC
*Mj*β-arrestin2-N-F	TACTCAGAATTCGTCAGTATGCCTACAAATCTA
*Mj*β-arrestin2-N-R	TACTCACTCGAGCTGGATTGGATGCTGTGC
**RNA interference**	
ds*Mj*SRC-F	GCGTAATACGACTCACTATAGGCACAGCCAGCAACTAAACC
ds*Mj*SRC-R	GCGTAATACGACTCACTATAGGCCGAACTCTGCGAGATGAA
ds*Mj*β-arrestin2-F	GCGTAATACGACTCACTATAGGGTTGCCTGTGGTGTAAGA
ds*Mj*β-arrestin2-R	GCGTAATACGACTCACTATAGGTCACGCAGAGGAATAGAG
ds*Mj*clathrin-F	GCGTAATACGACTCACTATAGGCCCAACGCTGGTTATGCT
ds*Mj*clathrin-R	GCGTAATACGACTCACTATAGGTGACCTGACCGCCTCTAC
dsGFP-F	GCGTAATACGACTCACTATAGGTGGTCCCAATTCTCGTGGAAC
dsGFP-R	GCGTAATACGACTCACTATAGGCTTGAAGTTGACCTTGATGCC

### RNA, genomic DNA extraction and cDNA synthesis

Total RNA was extracted from different tissues of shrimp using the TRIzol (Invitrogen, Carlsbad, CA, USA). The genomic DNA from shrimp tissues was extracted using a genomic DNA extraction kit (Toyobo, Osaka, Japan), according to the manufacturer’s instructions. First strand cDNA synthesis was performed using a cDNA Synthesis Kit (M-MLV version; Takara, Dalian, China), following the manufacturer’s instructions.

### cDNA cloning and sequence analysis of *Mj*SRC

The full-length cDNA sequence of a scavenger receptor C was obtained from hemocyte transcriptomic sequencing of *M*. *japonicus* (BGI, Shenzhen, China) and designated as *Mj*SRC. A pair of primers (*Mj*SRC-ORF-F and *Mj*SRC-ORF-R; Table 1) was used to amplify the ORF cDNA of *Mj*SRC for sequence confirmation. The *Mj*SRC sequence was translated conceptually and the deduced protein was predicted using ExPASy (http://cn.expasy.org/). Similarity analysis was conducted using BLAST (http://blast.ncbi.nlm.nih.gov/Blast.cgi/) and the domain architecture prediction of the proteins was performed using SMART (http://smart.embl-heidelberg.de). A phylogenetic analysis of *Mj*SRC was conducted using the MEGA 5.05 program [65].

### Recombinant expression, purification, and antiserum production

The fragment encoding the extracellular domains of *Mj*SRC was amplified using specific primers *Mj*SRC-EX-F and *Mj*SRC-EX-R (Table 1). The PCR procedure was as follows: one cycle at 95°C for 3 min; 35 cycles at 94°C for 30 s, 53°C for 30 s, and 72°C for 45 s; and one cycle at 72°C for 10 min. Afterwards, the purified PCR product was digested with restriction enzymes *Eco*RI and *Not*I and then ligated into the pET-32a(+) vector (GE Healthcare, Piscataway, NJ, USA). The recombinant plasmid was transformed into *E*. *coli* Rosseta (DE3) cells. Protein expression was induced with 0.5 mM isopropyl-β-D-thiogalactopyranoside (IPTG). The proteins were expressed as inclusion bodies, denatured in 8 M urea and purified using Ni-NTA beads. The proteins were then refolded in a buffer containing 50 mM Tris-HCl, 50 mM NaCl, 0.5 mM EDTA, 1% glycine, and 10% glycerol at pH 7.9 and dialyzed in PBS overnight. The refolded purified proteins were used for subsequent experiments. Rabbit antiserum against *Mj*SRC was prepared as previously described [66].

### Tissue distribution and expression profiles of *Mj*SRC

The tissue distribution and temporal expression of *Mj*SRC mRNA were determined by qRT-PCR using primers *Mj*SRC-RT-F and *Mj*SRC-RT-R (Table 1). The qRT-PCR was carried out using SYBR Premix Ex Taq (Takara), following the manufacturer’s instructions, and conducted in a real-time thermal cycler (Bio-Rad, Hercules, CA, USA). The total volume was 10 μL, including 5 μL of Premix Ex Taq, 2 μL of each primer (1 mM) and 1 μL of cDNA (1:50 diluted). β-Actin-RT-F and β-actin-RT-R primers (Table 1) were used to amplify *β-actin*, which served as the internal control. The data obtained were calculated using the cycle threshold (2^−ΔΔCT^) method, as previously described [67]. To study the temporal expression of *Mj*SRC, different tissues were collected from shrimp at 0, 3, 6, 12, 24, 36, 48 and 72 h after challenged with WSSV. RNA extraction and reverse transcription were performed as above. After qRT-PCR analysis, the data obtained were presented as the mean ± SD from three independent experiments and analyzed statistically using Student’s *t*-test. Significant differences were accepted at *p* < 0.05

The distribution and expression profiles of the *Mj*SRC protein were evaluated by western blotting. Briefly, the tissues were homogenized in radio-immunoprecipitation assay (RIPA) buffer (50 mM Tris-HCl, 150 mM NaCl, 0.1% SDS, 0.5% Nonidet P-40, 1 mM EDTA, 0.5 mM PMSF, pH 8.0). The homogenate was centrifuged at 12,000 × g for 10 min at 4°C, and the supernatant was collected. After separation by 12.5% SDS-PAGE, proteins were transferred onto a nitrocellulose membrane, and blocked with 5% nonfat milk diluted in TBS (10 mM Tris-HCl, 150 mM NaCl, pH 8.0) for 1 h with gentle shaking. The membrane was then incubated with primary antiserum against *Mj*SRC (diluted 1:200 in the blocking milk solution) overnight at 4°C. After three washes with TBST (TBS containing 0.1% Tween-20), the membrane was incubated with HRP-conjugated goat anti-rabbit antibody (ZSGB Bio, Beijing, China, 1:10,000 dilution in blocking reagent) for 3 h at room temperature with gentle shaking. The membrane was then washed with TBST three times for 10 min each, and the target bands were visualized by a solution of 1 mL 4-chloro-1-naphthol and 6 μL H_2_O_2_ in 10 mL TBS in the dark. The expression of β-actin was used to normalize the amounts of loaded proteins.

### RNA interference and overexpression

To knockdown the expression of *Mj*SRC, an RNA interference assay was performed by injecting double-strand RNA (*dsMjSRC*). Primers ds*Mj*SRC-F and ds*Mj*SRC-R (Table 1), incorporating a T7 promoter, were used to amplify the template to produce the dsRNA (about 500 bp) (Fig 2A) with T7 RNA polymerase (Fermentas, Burlington, Canada). Green fluorescent protein (GFP) served as the control and *dsGFP* RNA was synthesized in the same way. Then, *dsMjSRC* and *dsGFP* RNA (50 μg each) were injected into the abdominal segment of shrimp and another 50 μg RNA was injected 12 h later. After 24 hours, the RNA interference efficiency in hemocytes at the mRNA and protein level was detected by qRT-PCR and western blotting, respectively. β-Actin was used as the internal reference.

We also performed overexpression of *Mj*SRC using *Mj*SRC mRNA. The ORF of *Mj*SRC was amplified using primers *Mj*SRC-ORF-F and *Mj*SRC-ORF-R (Table 1) and then ligated into the pET-32a(+) vector containing a T7 promoter. The recombinant plasmid was used as a template to transcribe the single stranded and capped *Mj*SRC mRNA (Fig 2A) using a T7 RNA polymerase *in vitro* transcription kit (Ambion, Inc. Austin, Texas, United States of America), according to the manufacturer’s instructions. The empty pET-32a (+) vector was used as a template to synthesize *Trx (Thioredoxin)-His tag* mRNA as a control. Each shrimp was injected with 100 μg *MjSRC* (or *Trx-His tag*) mRNA and at least three shrimp were used in each group. After 24 h of mRNA injection, the efficiency of overexpression was analyzed by western blotting using an anti-*Mj*SRC antibody in the hemocytes of each group.

RNA interference of *Mj*β-arrestin2 and *Mj*clathrin used primers ds*Mj*β-arrestin2-F/R and ds*Mj*clathrin-F/R (Table 1) and followed the same method as described above.

### Fluorescent labeling of WSSV and detection of the phagocytic rate and phagocytic index *in vivo*

Fluorescein isothiocyanate (FITC; Sigma-Aldrich, St. Louis, MO, USA) was used to label WSSV for 1.5 h at 37°C. The detailed procedure was performed according to previous reports [68, 69]. After 24 h of RNAi or overexpression of *Mj*SRC, shrimp were injected with FITC-labeled WSSV (1×10^5^ virions). One hour after WSSV injection, hemocytes were collected and spread on the slides. The hemocytes were then fixed in 4% paraformaldehyde for 10 min at room temperature (RT), washed three times with PBS and observed under a fluorescence microscope (Olympus BX51, Japan). The phagocytic rate was defined as [the hemocytes engulfing WSSV / all hemocytes observed] × 100%. The phagocytotic index of hemocytes was defined as (virion numbers in all hemocytes / all hemocytes observed) × 100%. To confirm the results, flow cytometry was performed using the CELL Quest program (Becton Dickinson, San Jose, CA, United States of America), following a previous report [31]. The data were presented as mean ± SD from three independent experiments and analyzed statistically using Student’s *t*-test. Significant differences were accepted at *p* < 0.05.

### Immunocytochemistry

To detect the subcellular localization of *Mj*SRC in hemocytes, immunocytochemistry was performed. The hemolymph was extracted from WSSV-infected shrimp and normal shrimp, centrifuged at 800 × g for 10 min at 4°C to collect hemocytes, washed twice with PBS, spread onto slides, fixed with 4% paraformaldehyde (diluted in PBS) and permeabilized with 0.2% triton X-100. After washing three times, the hemocytes were blocked with 3% bovine serum albumin (diluted in PBS) for 1 h at 37°C, and then incubated with anti-*Mj*SRC antibody (1:100, diluted in blocking reagent) overnight at 4°C. On the following day, the slides were washed with PBS six times, and then incubated with 1:1000 diluted goat anti-rabbit-ALEXA 488 (Molecular Probes) for 1 h at 37°C. The cell nuclei were stained with 4-6-diamidino-2-phenylindole (DAPI) for 10 min. After washing six times with PBS, the slides were observed under the fluorescence microscope.

The co-localization of *Mj*SRC and FITC labeled-WSSV in shrimp hemocytes was also detected. WSSV was labeled with FITC (green) and injected into shrimp. Hemocytes were collected at different time points (15, 30, 45 and 60 min) after WSSV injection. The second antibody for detecting *Mj*SRC is anti-rabbit IgG Alexa-546 (red). Nuclei were stained with DAPI (blue).

### Pull-down and co-immunoprecipitation assays

Pull-down assays were performed to explore whether recombinant *Mj*SRC (r*Mj*SRC) could interact with the main envelope proteins of WSSV (VP19, VP24, VP26 and VP28). The primers for recombinant expression of VPs (VP-F and VP-R) are shown in Table 1. The amplified sequences of VP19, VP24, VP26 and VP28 were ligated separately into vector pET32A and transformed into *E*. *coli* Rosseta for expression. *Mj*SRC comprises the extracellular region (*Mj*SRC-EX) and intracellular region (*Mj*SRC-IN); *Mj*SRC-EX contains two different domains: the complement control protein (CCP) domain and domain in meprin, A5, receptor protein tyrosine phosphatase mu (MAM). These different domains were amplified separately using the primers listed in Table 1, and designated as *MjSRC-EX*, *MjSRC-CCP*, *MjSRC-MAM* and *MjSRC-IN*. They were ligated separately into vector pGEX-4T-1 (GE Healthcare), and transformed into *E*. *coli* Rosseta. The recombinantly expressed proteins were purified by affinity chromatography using GST-resin (GenScript, Nanjing, China). Two hundred microliters of r*Mj*SRC and 200 μL of rVPs protein solutions (1 μg/μL, diluted in TBS) were incubated at 4°C for 30 min and then GST-bind resin (20 μL) was added. The resin was washed with TBS thoroughly, the proteins were eluted with elution buffer (10 mM reduced glutathione and 50 mM Tris-HCl, pH 8.0) and then analyzed using 12.5% SDS-PAGE.

Co-IP was performed to confirm the interaction between *Mj*SRC and VP proteins. The hemocytes from virus-infected shrimp were lysed in RIPA buffer and centrifuged at 12,000 × g for 10 min at 4°C. The supernatant (about 500 μL, 1 mg/mL) was incubated with 20 μL protein A beads with gentle shaking at 4°C for 30 min to remove non-specific binding. After centrifugation at 3000 × g for 5 min, the supernatant was incubated with 50 μL antiserum of *Mj*SRC or VP19 at 4°C overnight. Protein A beads (30 μL) were then added to the mixture and incubation continued for 1 h at 4°C. After centrifugation at 3000 × g for 5 min, the beads were collected and washed with PBS three times. Subsequently, the pellets were suspended in 40 μL 1 × electrophoresis sample buffer and denatured at 95°C for 5 min, followed by SDS-PAGE and western blotting using antiserum for VP19 or *Mj*SRC. Normal rabbit IgG was used as a control. The interaction between *Mj*SRC-IN with *Mj*β-arrestins was also analyzed similarly using pull-down and co-IP assays.

### Colloidal gold labeling of *Mj*SRC and transmission electron microscopy

To investigate whether *Mj*SRC could bind to WSSV, immunoelectron microscopy was performed following the procedure detailed in our previous report [41]. Briefly, r*Mj*SRC-EX was labeled with gold nanoparticles (diameter of 10 nm, Sigma, USA). The pH of the colloidal gold was adjusted to be 0.5 higher than the isoelectric point (pI) of the r*Mj*SRC-EX protein solution. Approximately 0.2 mL of colloidal gold was added into 0.2 mL of the protein solution (1 mg/mL), and the mixture was incubated for 20 min at 4°C with gentle shaking. After adding 1% polyethylene glycol (PEG) to a final concentration of 0.04%, the mixture was centrifuged at 50,000 × g for 45 min. The precipitate was resuspended with 0.2 mL PBS containing 0.04% PEG and then stored at 4°C. The tag protein Trx-His tag was used as a control, and was also labeled with gold nanoparticles. The virions of WSSV were purified by a previously reported method [70], and the purified virions were absorbed onto carbon-coated nickel grids, and incubated with labeled r*Mj*SRC-EX (or Trx-His tag) for 10 min at RT. After washing with distilled water three times, the samples were counterstained with 2% sodium phosphotungstate (Zhongjingkeyi Technology Co., Ltd, Beijing, China) for 30 seconds and finally observed under a transmission electron microscope (EM-100CXII).

### Crosslinking assay

Suberic acid bis (3-sulfo-N-hydroxysuccinimide ester) sodium salt (BS3; Sigma-Aldrich) possesses a charged group and is useful for cell-surface protein crosslinking. The assay was performed according to the manufacturer’s protocol. Briefly, hemocytes from shrimp were collected and washed three times with ice-cold PBS (pH 8.0). BS3 was added to the resuspended hemocytes to a final concentration of 5 mM and the reaction mixture was incubated at 4°C for 1 h. Quenching buffer (Tris-HCl, pH 7.5) was then added into the mixture with a final concentration of 20 mM to quench the crosslinking reaction. The crosslinking-treated hemocytes were homogenized and subjected to western blotting.

### Clathrin inhibitor assay

Chlorpromazine (CPZ, Sangon Biotech, Shanghai, China,) was used to inhibit the phagocytosis of WSSV in shrimp. CPZ prevents recycling of clathrin to the plasma membrane thereby inhibiting clathrin-mediated endocytosis [71, 72]. CPZ was diluted in sterile water at 10 μg/μL, and different amounts of CPZ solution were injected into shrimp. In our experiment, WSSV inoculums were injected into shrimp 24 h before CPZ injection. The amount of WSSV in the shrimp was detected via RT-PCR and western blotting using VP28 as a marker. Furthermore, the effect of CPZ on phagocytosis of hemocytes was also detected. After 1 h of CPZ injection, FITC-labeled WSSV was injected into the shrimp and 1 h later, hemocytes were collected and the phagocytic rate was calculated. Sterile water injection was used as the control.

### Lysosome staining and lysosome inhibitor injection

Lysosome staining kit (Bio Basic Inc., Markham, Ontario, Canada) was used to stain lysosomes in hemocytes following the manufacturer’s protocol. Briefly, hemocytes were collected from three shrimp and spread onto slides. After washed with PBS, the hemocytes were stained with LysoBrite Red (1:500 diluted in live cell staining buffer) for 1 h at room temperature. Then washed three times with PBS, hemocytes were stained with DAPI to label cell nuclei. The slides were observed under the fluorescence microscope.

Chloroquine (CLQ, Sangon Biotech, China), a lysosome inhibitor, was used to inhibit the lysosome function. CLQ was used at 10 mg/kg to inhibit the function of lysosome in mouse [73]. In this study, CLQ was diluted in PBS at 10 μg/μL, and different volume of CLQ solution (0, 2.5, 7.5 and 10 μL) was injected into shrimp. The amount of WSSV in the shrimp was detected via RT-PCR and western blotting using VP28 as a marker.

## Supporting Information

S1 Fig Schematic representation of SRCs. Several SRCs are from *Bombyx mori* (NP001128387), *Spodoptera frugiperda* (ABB92836), *Aedes aegypti* (AAEL006361), *Anopheles darlingi* (ETN63673), *Drosophila melanogaster* (AGA18734) and *Marsupenaeus japonicus* (KU213605). The functional modules were predicted in SMART (http://smart.embl-heidelberg.de/).
(TIF)

S2 Fig Phylogenetic analysis of SRCs from *M*. *japonicus* and other species. The neighbor-joining tree was constructed by MEGA 5.05, using bootstraps of 1000 to test the reproducibility. *Mj*SRC is labeled with a black triangle. The GenBank accession number of each sequence is shown in the figure. *A*. *darlingi*: *Anopheles darlingi*; *B*. *mori*: *Bombyx mori*; *C*. *quinquefasciatus*: *Culex quinquefasciatus*; *D*. *plexippus*: *Danaus plexippus*; *D*. *melanogaster*: *Drosophila melanogaster*; *D*. *simulans*: *Drosophila simulans*; *D*. *yakuba*: *Drosophila yakuba*; *Mj*: *Marsupenaeus japonicus*; *R*. *pedestris*: *Riptortus pedestris*; *S*. *frugiperda*: *Spodoptera frugiperda*.
(TIF)

S3 Fig The expression profiles of *Mj*SRC in gills and intestine of shrimp challenged by WSSV. (A and B) The mRNA expression patterns of *Mj*SRC in gills (A) and intestine (B) of shrimp after WSSV challenge detected by qRT-PCR with β-actin gene as a reference. Results were expressed as the mean ± SD and analyzed statistically by student’s *t*-test. (C) The protein expression patterns of *Mj*SRC in hemocytes of shrimp after WSSV challenge, detected by western blotting with β-actin as the reference. The experiments were repeated three times independently, and were used for bands scanning with Quantity One software in Fig 1D. (D) VP28 protein levels in hemocytes of shrimp after WSSV challenge analyzed by western blotting. β-Actin was used as the sample loading control.
(TIF)

S4 Fig *Mj*SRC restricts WSSV replication in shrimp. (A) Efficiency of *Mj*SRC RNAi in hemocytes at different time points, as detected by RT-PCR (upper two panels) and western blotting (lower two panels). The β-actin gene served as the reference. (B) Efficiency of *Mj*SRC overexpression in hemocytes, as detected by western blotting with anti-*Mj*SRC sera. *Trx-His tag* mRNA overexpression was used as the control. (C) WSSV replication in shrimp after overexpression of *Mj*SRC. The shrimp was injected with WSSV after *MjSRC* mRNA injection. The amounts of virions were determined at 60 h after WSSV injection using western blotting.
(TIF)

S5 Fig pBlueScript II SK+ with *VP28* sequence was used to generate standard curve by qRT-PCR for WSSV quantification. (A and B) Amplification curves (A) and melting curves (B) generated by qRT-PCR to amplify *VP28* fragments using different quantified plasmids as templates. The value of melting temperature was 85°C. (C) Standard curve generated using above qRT-PCR data. Log Quantity, the log of the template copy number; C(T) Cycle, PCR cycle number.
(TIF)

S6 Fig Hemocyte phagocytosis of WSSV observed under a fluorescence microscope. After knockdown or overexpression of *Mj*SRC, FITC labeled WSSV (green) were injected into shrimp. Hemocytes were collected at 1 h after WSSV injection. Nuclei were stained with DAPI (blue). Scale bar = 100 μm. The last column showed local amplification of parts in the box. *dsGFP* and *Trx-His tag* mRNA were used as the control.
(TIF)

S7 Fig Serial photographs of hemocyte phagocytosis of WSSV observed under the flow cytometry after *dsGFP* (A) or *dsMjSRC* (B) injection. Ch 01: channel 01, bright field images of hemocytes; Ch 02: channel 02, FITC-labeled WSSV images; Ch 03: channel 03, the merged images of Ch 01 and Ch 02, indicating WSSV located in hemocytes.
(TIF)

S8 Fig Oligomerization of *Mj*SRC in hemocytes of shrimp infected by WSSV. (A) A trimer of *Mj*SRC was detected *in vivo* using western blotting after treatment with crosslinker (BS3). Western blotting was performed using anti-*Mj*SRC sera. β-Actin served as loading control. (B) Hemocytes were collected from WSSV-infected shrimp and treated with different concentration of BS3. These hemocytes were homogenized, separated by SDS-PAGE and detected with western blotting.
(TIF)

S9 Fig *Mj*β-arrestin2 was upregulated in hemocytes of shrimp challenged by WSSV. (A and B) Tissue distribution of *Mj*β-arrestin1 (A) and *Mj*β-arrestin2 (B) in shrimp. The mRNA expression level was analyzed by qRT-PCR. β-Actin was used as an internal reference. (C and D) mRNA expression patterns of *Mj*β-arrestin1 (C) and *Mj*β-arrestin2 (D) in hemocytes of shrimp after WSSV challenge detected by qRT-PCR with β-actin gene as a reference. Results were expressed as the mean ± SD and analyzed statistically by student’s *t*-test.
(TIF)

S10 Fig Phylogenetic analysis of clathrins from *M*. *japonicus* and other species. The neighbor-joining tree was produced by MEGA 5.05, using bootstraps of 1000 to test the reproducibility. *Mj*clathrin is labeled with a black triangle. The GenBank accession number of each sequence is shown in the figure. *A*. *echinatior*: *Acromyrmex echinatior*; *B*. *mori*: *Bombyx mori*; *D*. *melanogaster*: *Drosophila melanogaster*; *D*. *plexippus*: *Danaus plexippus*; *H*. *sapiens*: *Homo sapiens*; *L*. *migratoria*: *Locusta migratoria*; *L*. *niger*: *Lasius niger*; *Mj*: *Marsupenaeus japonicus*; *M*. *musculus*: *Mus musculus*; *P*. *humanus corporis*: *Pediculus humanus corporis*; *P*. *monodon*: *Penaeus monodon*; *R*. *norvegicus*: *Rattus norvegicus*; *R*. *pedestris*: *Riptortus pedestris*; *S*. *mimosarum*: *Stegodyphus mimosarum*; *T*. *castaneum*: *Tribolium castaneum*; *Z*. *nevadensis*: *Zootermopsis nevadensis*.
(TIF)
