# Supplementary figures and images for: Scavenger Receptor C Mediates Phagocytosis of White Spot Syndrome Virus and Restricts Virus Proliferation in Shrimp
Source: PLoS Pathog. 2016 Dec 27;12(12):e1006127. doi: 10.1371/journal.ppat.1006127 (PMC5222524; doi:10.1371/journal.ppat.1006127)

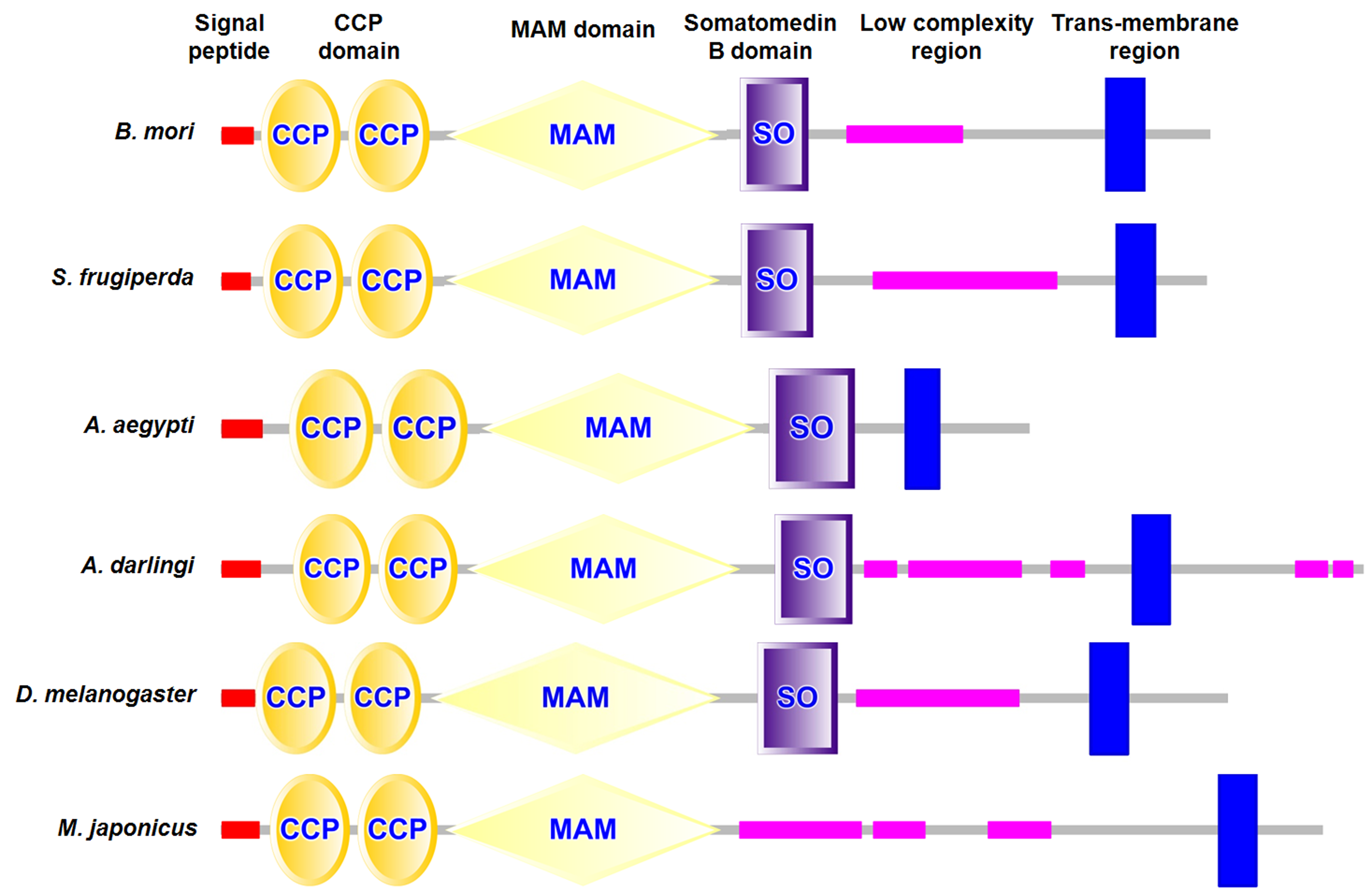

Supplement: S1 Fig — Several SRCs are from Bombyx mori (NP001128387), Spodoptera frugiperda (ABB92836), Aedes aegypti (AAEL006361), Anopheles darlingi (ETN63673), Drosophila melanogaster (AGA18734) and Marsupenaeus japonicus (KU213605). The functional modules were predicted in SMART (http://smart.embl-heidelberg.de/). (TIF) [file ppat.1006127.s001.tif]

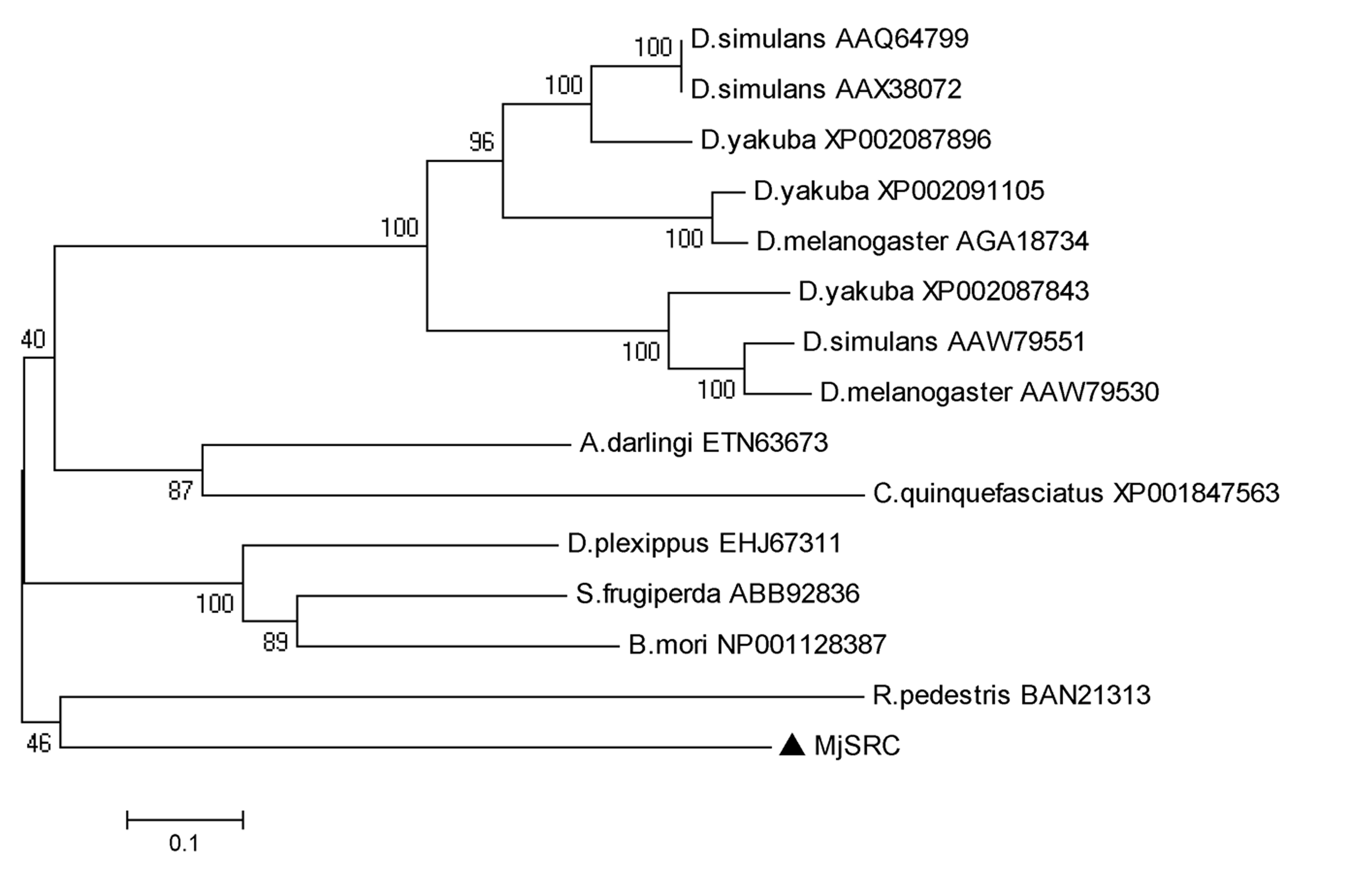

Supplement: S2 Fig — The neighbor-joining tree was constructed by MEGA 5.05, using bootstraps of 1000 to test the reproducibility. MjSRC is labeled with a black triangle. The GenBank accession number of each sequence is shown in the figure. A. darlingi: Anopheles darlingi; B. mori: Bombyx mori; C. quinquefasciatus: Culex quinquefasciatus; D. plexippus: Danaus plexippus; D. melanogaster: Drosophila melanogaster; D. simulans: Drosophila simulans; D. yakuba: Drosophila yakuba; Mj: Marsupenaeus japonicus; R. pedestris: Riptortus pedestris; S. frugiperda: Spodoptera frugiperda. (TIF) [file ppat.1006127.s002.tif]

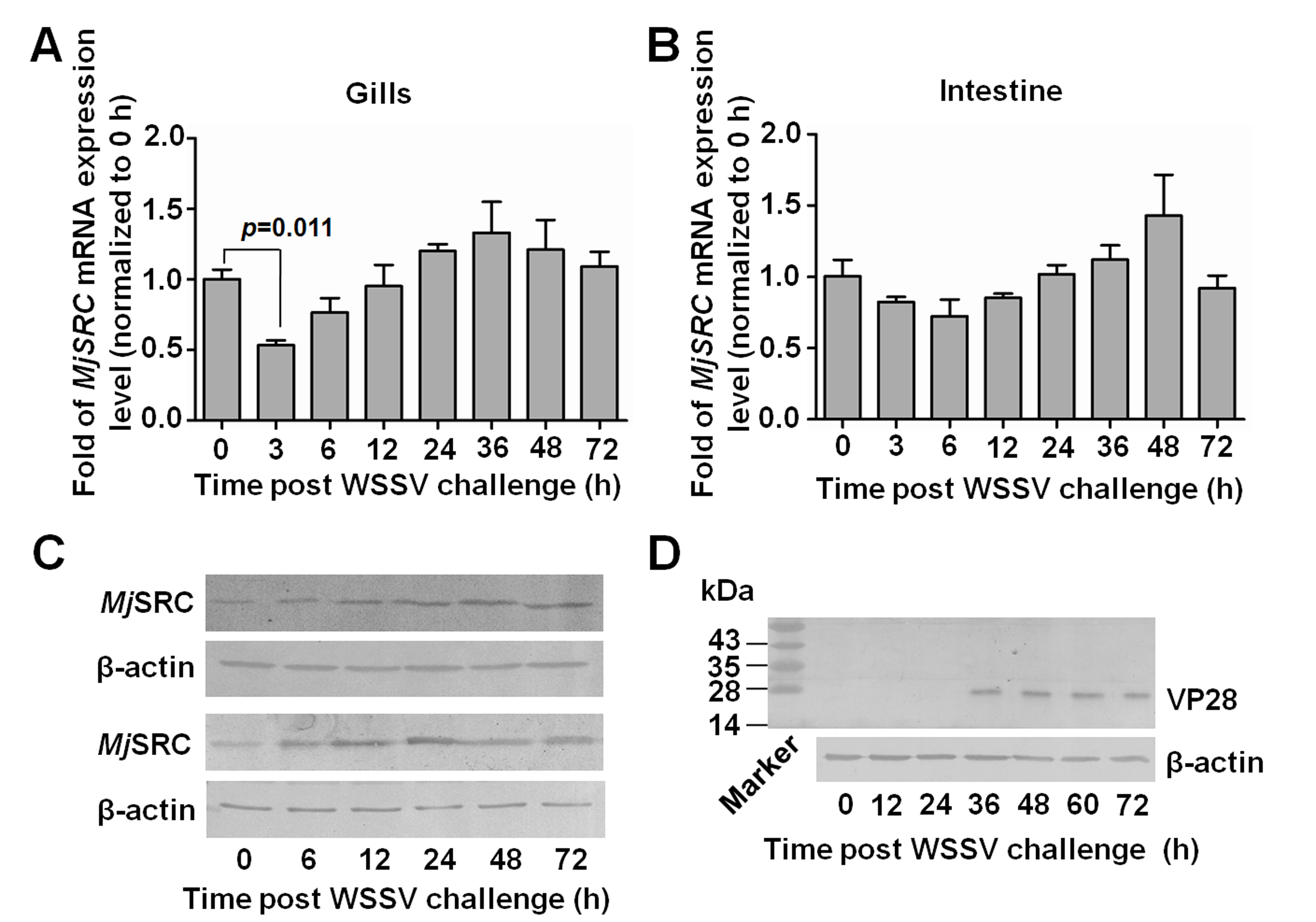

Supplement: S3 Fig — (A and B) The mRNA expression patterns of MjSRC in gills (A) and intestine (B) of shrimp after WSSV challenge detected by qRT-PCR with β-actin gene as a reference. Results were expressed as the mean ± SD and analyzed statistically by student’s t-test. (C) The protein expression patterns of MjSRC in hemocytes of shrimp after WSSV challenge, detected by western blotting with β-actin as the reference. The experiments were repeated three times independently, and were used for bands scanning with Quantity One software in Fig 1D. (D) VP28 protein levels in hemocytes of shrimp after WSSV challenge analyzed by western blotting. β-Actin was used as the sample loading control. (TIF) [file ppat.1006127.s003.tif]

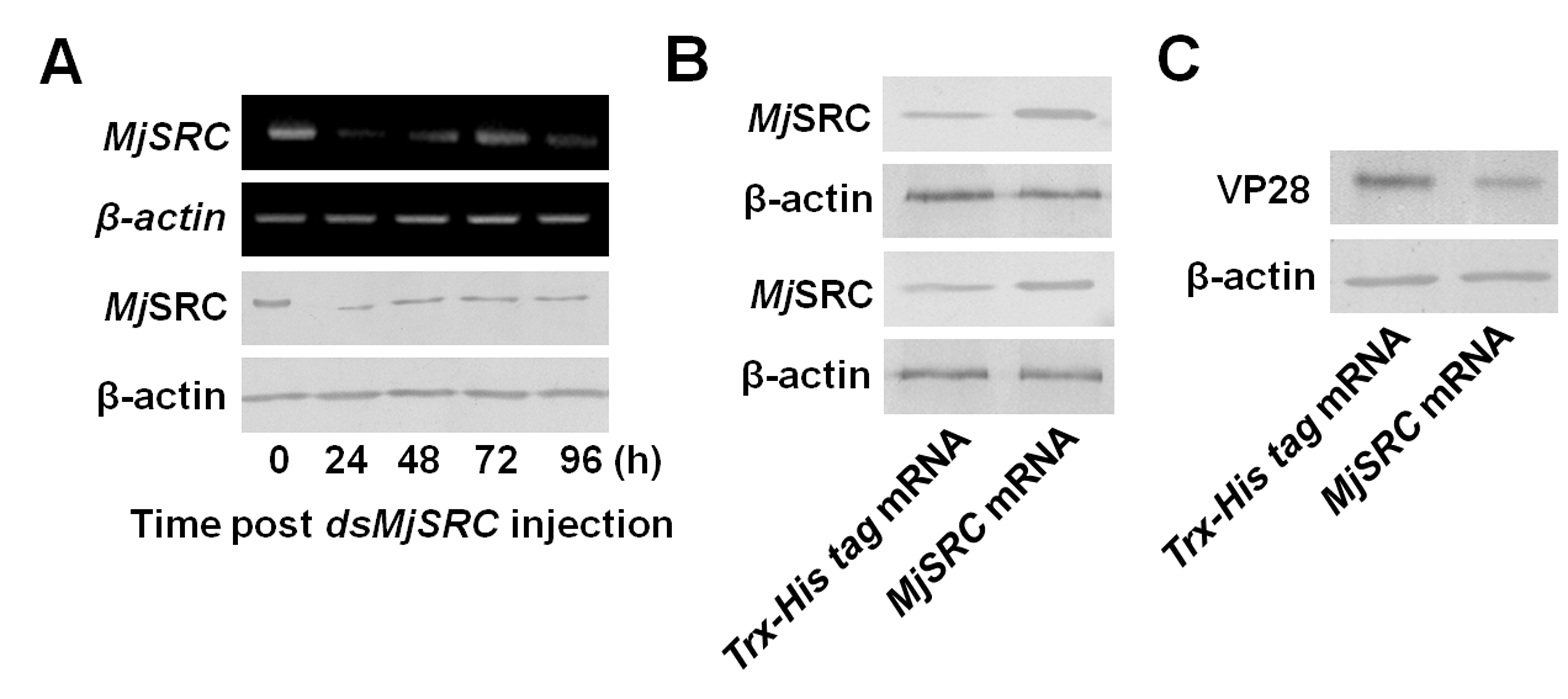

Supplement: S4 Fig — (A) Efficiency of MjSRC RNAi in hemocytes at different time points, as detected by RT-PCR (upper two panels) and western blotting (lower two panels). The β-actin gene served as the reference. (B) Efficiency of MjSRC overexpression in hemocytes, as detected by western blotting with anti-MjSRC sera. Trx-His tag mRNA overexpression was used as the control. (C) WSSV replication in shrimp after overexpression of MjSRC. The shrimp was injected with WSSV after MjSRC mRNA injection. The amounts of virions were determined at 60 h after WSSV injection using western blotting. (TIF) [file ppat.1006127.s004.tif]

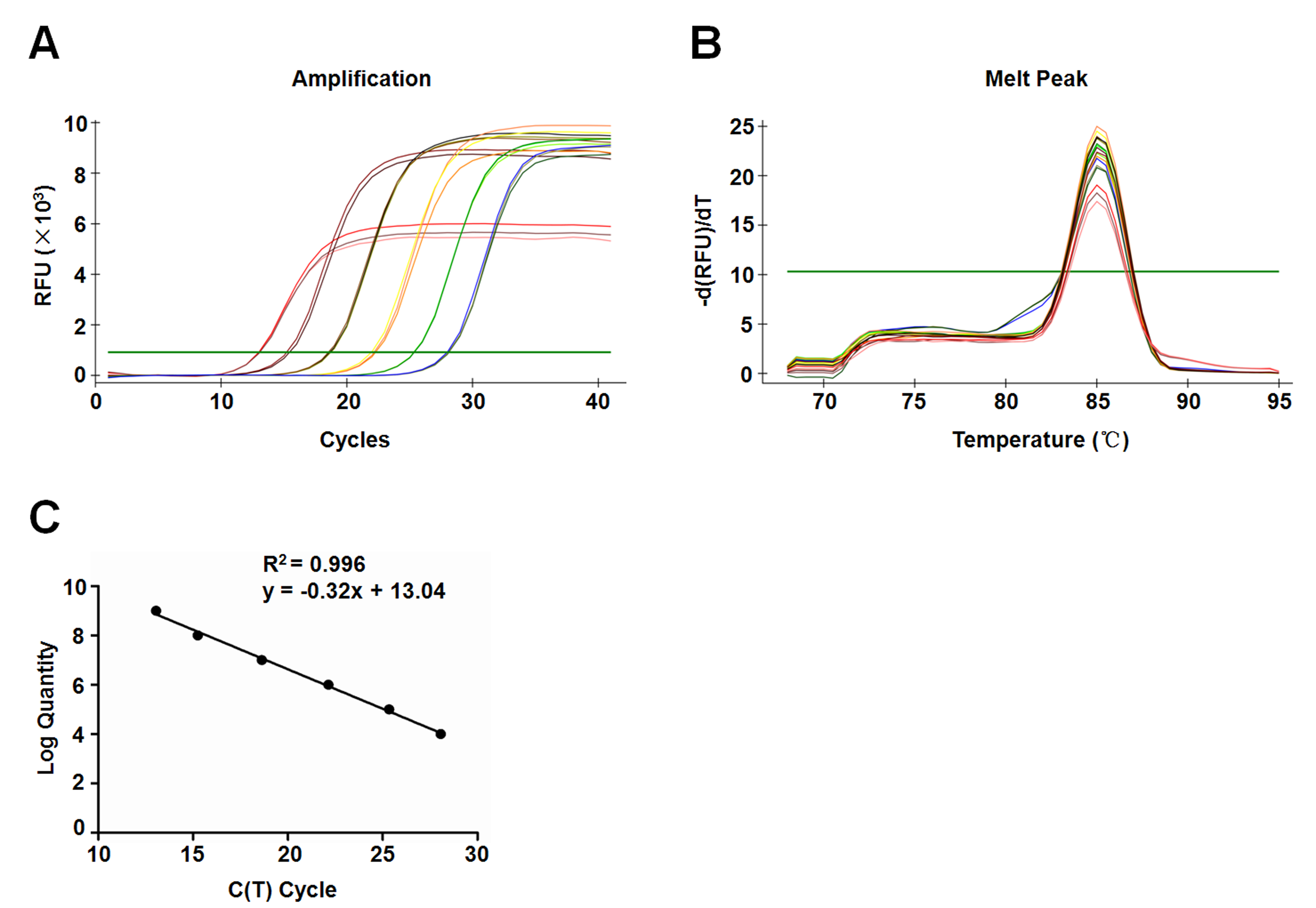

Supplement: S5 Fig — (A and B) Amplification curves (A) and melting curves (B) generated by qRT-PCR to amplify VP28 fragments using different quantified plasmids as templates. The value of melting temperature was 85°C. (C) Standard curve generated using above qRT-PCR data. Log Quantity, the log of the template copy number; C(T) Cycle, PCR cycle number. (TIF) [file ppat.1006127.s005.tif]

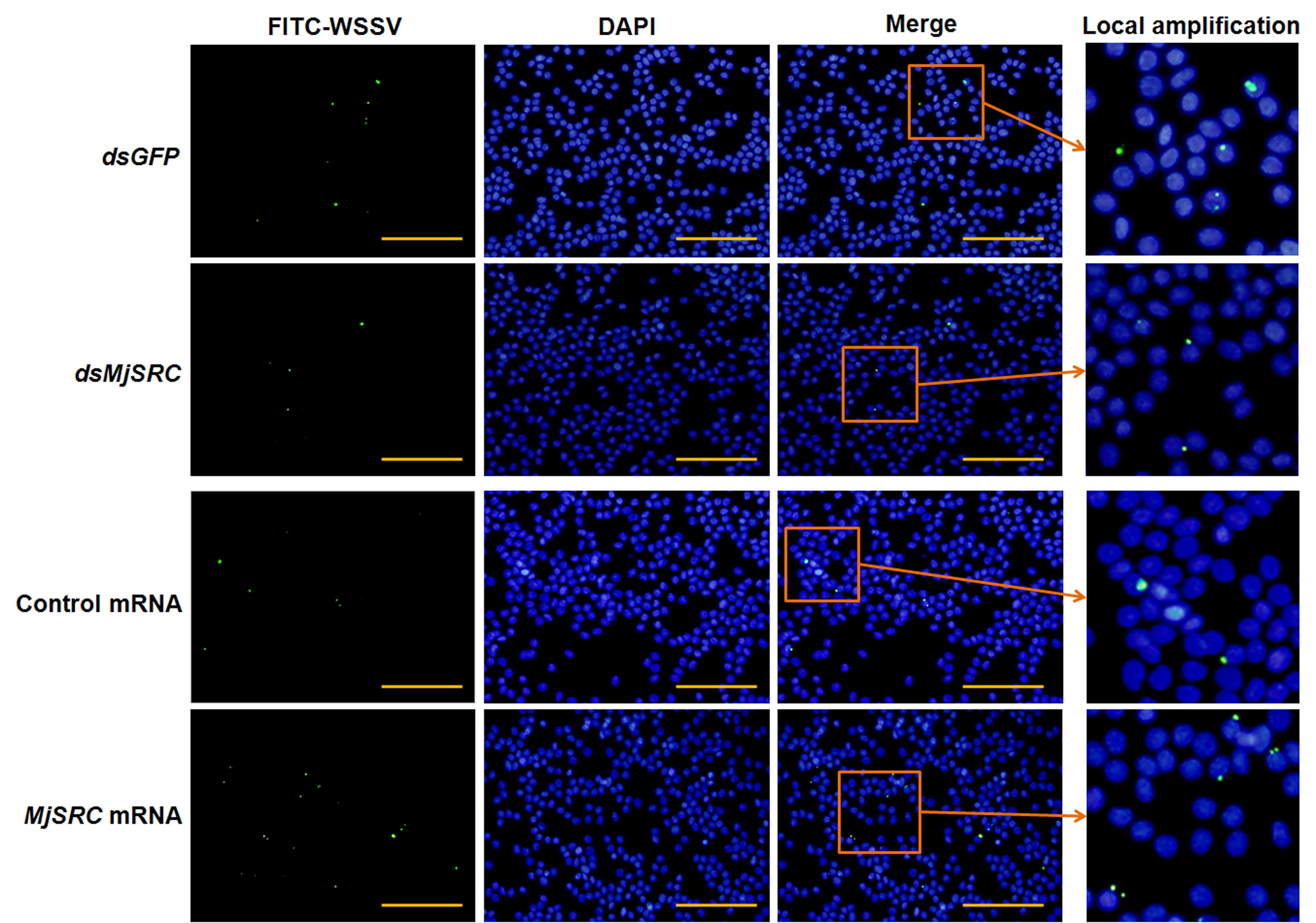

Supplement: S6 Fig — After knockdown or overexpression of MjSRC, FITC labeled WSSV (green) were injected into shrimp. Hemocytes were collected at 1 h after WSSV injection. Nuclei were stained with DAPI (blue). Scale bar = 100 μm. The last column showed local amplification of parts in the box. dsGFP and Trx-His tag mRNA were used as the control. (TIF) [file ppat.1006127.s006.tif]

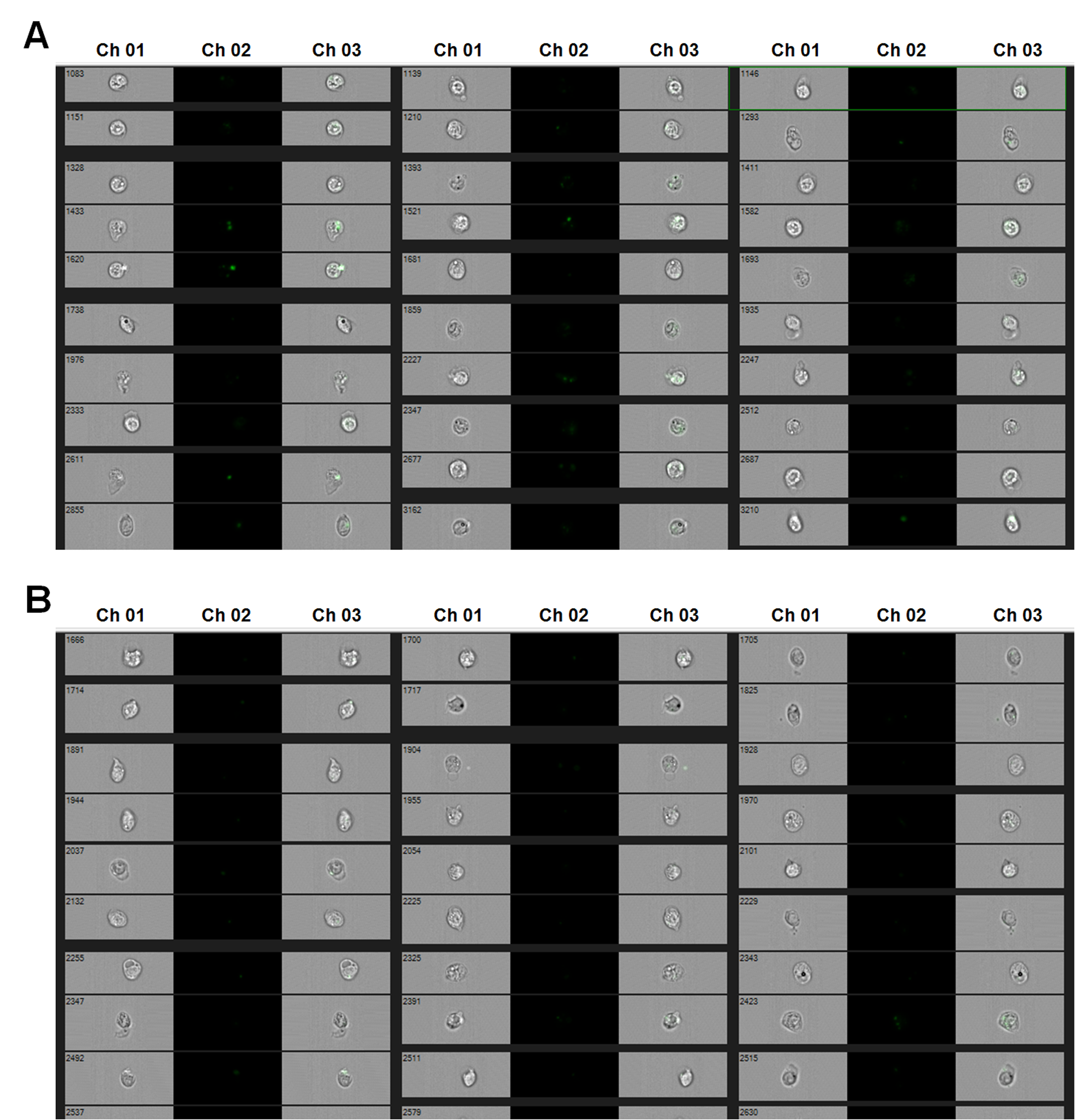

Supplement: S7 Fig — Ch 01: channel 01, bright field images of hemocytes; Ch 02: channel 02, FITC-labeled WSSV images; Ch 03: channel 03, the merged images of Ch 01 and Ch 02, indicating WSSV located in hemocytes. (TIF) [file ppat.1006127.s007.tif]

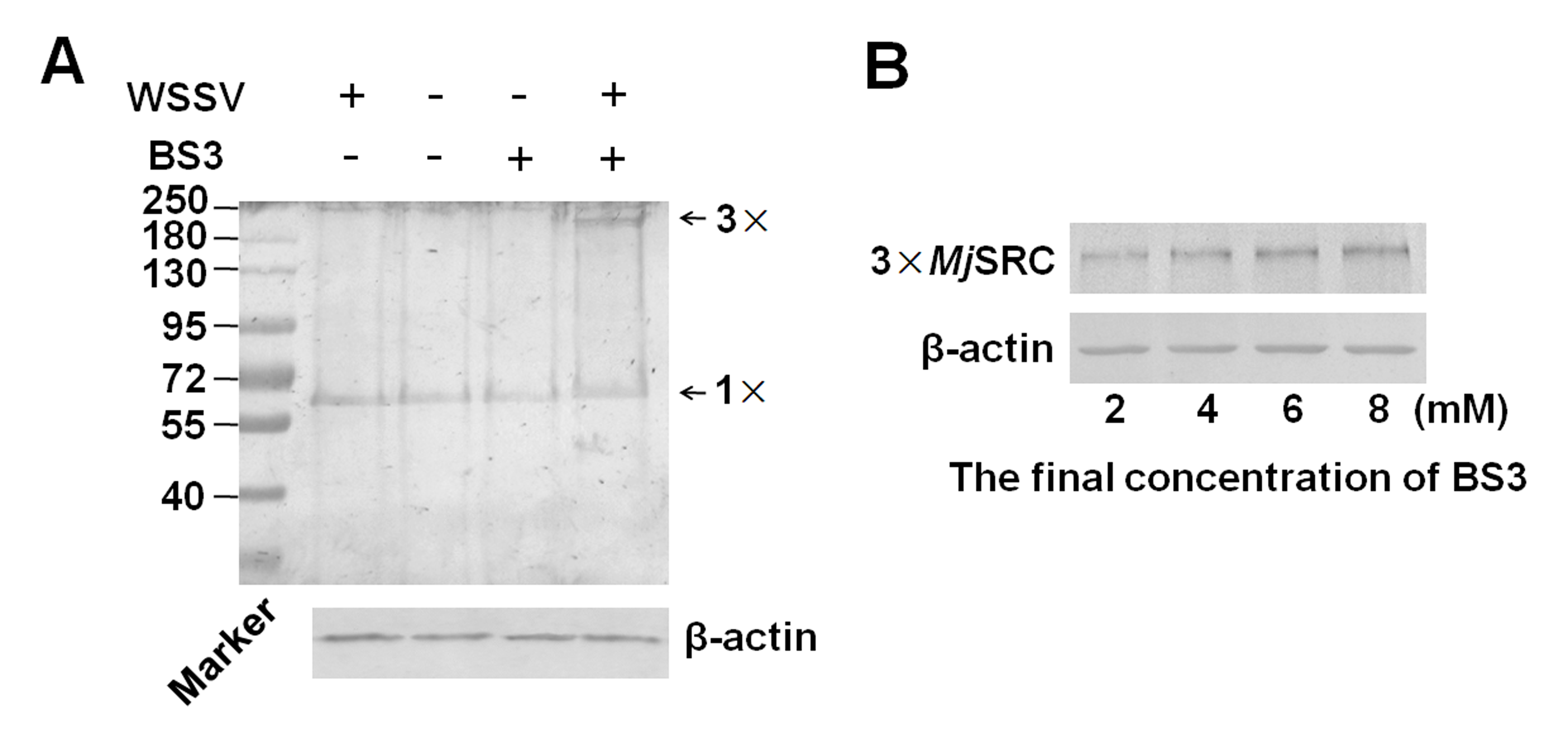

Supplement: S8 Fig — (A) A trimer of MjSRC was detected in vivo using western blotting after treatment with crosslinker (BS3). Western blotting was performed using anti-MjSRC sera. β-Actin served as loading control. (B) Hemocytes were collected from WSSV-infected shrimp and treated with different concentration of BS3. These hemocytes were homogenized, separated by SDS-PAGE and detected with western blotting. (TIF) [file ppat.1006127.s008.tif]

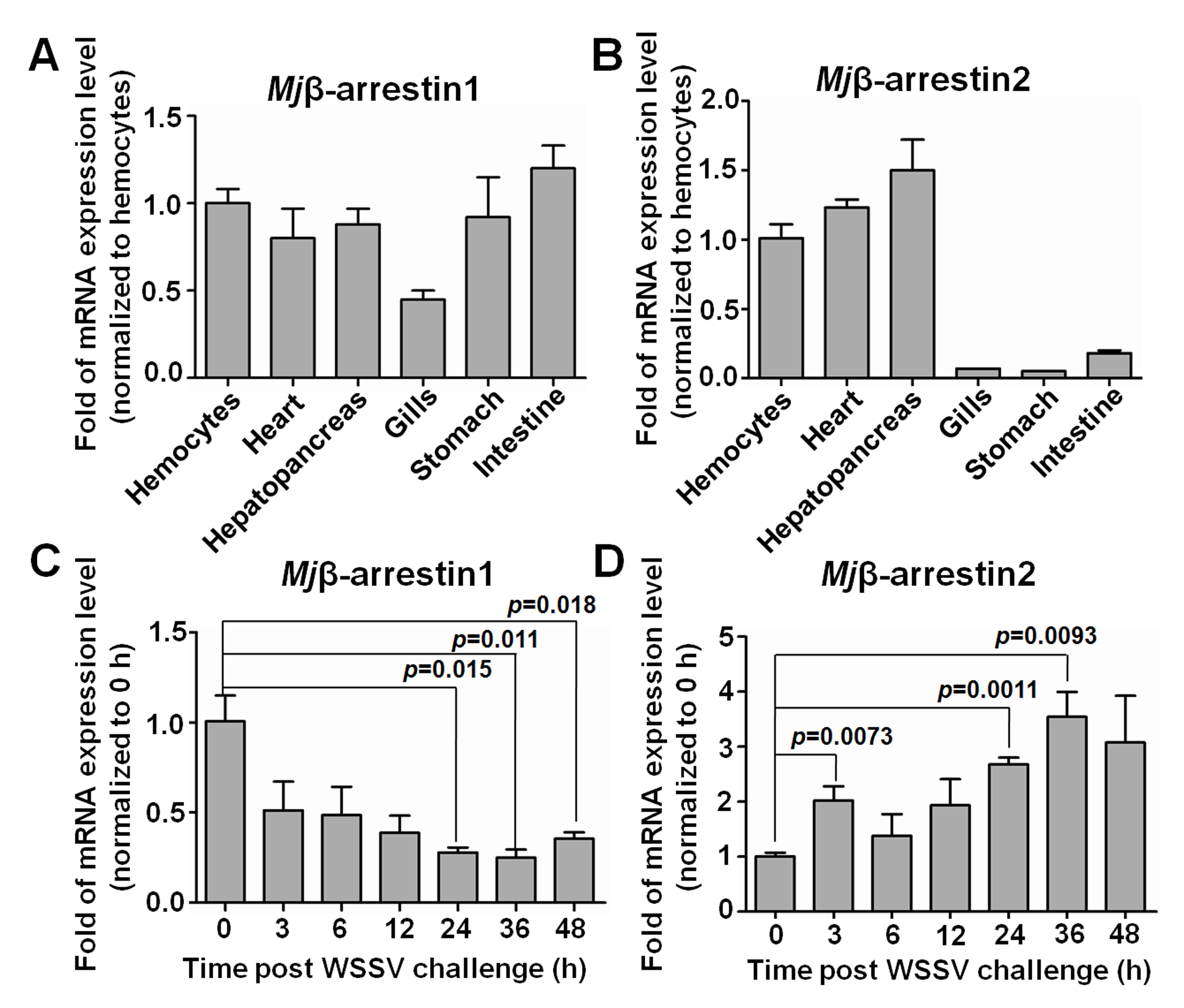

Supplement: S9 Fig — (A and B) Tissue distribution of Mjβ-arrestin1 (A) and Mjβ-arrestin2 (B) in shrimp. The mRNA expression level was analyzed by qRT-PCR. β-Actin was used as an internal reference. (C and D) mRNA expression patterns of Mjβ-arrestin1 (C) and Mjβ-arrestin2 (D) in hemocytes of shrimp after WSSV challenge detected by qRT-PCR with β-actin gene as a reference. Results were expressed as the mean ± SD and analyzed statistically by student’s t-test. (TIF) [file ppat.1006127.s009.tif]

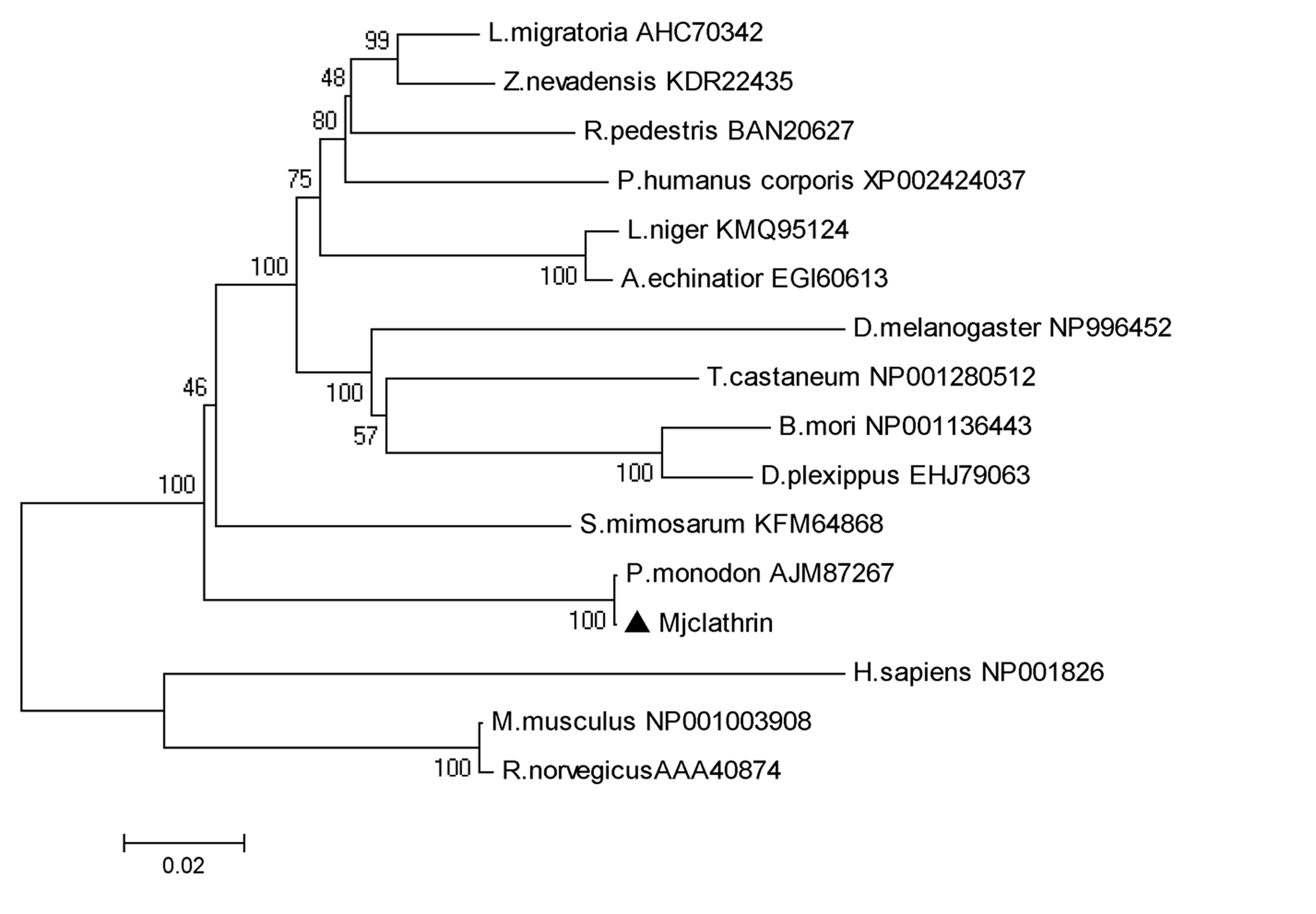

Supplement: S10 Fig — The neighbor-joining tree was produced by MEGA 5.05, using bootstraps of 1000 to test the reproducibility. Mjclathrin is labeled with a black triangle. The GenBank accession number of each sequence is shown in the figure. A. echinatior: Acromyrmex echinatior; B. mori: Bombyx mori; D. melanogaster: Drosophila melanogaster; D. plexippus: Danaus plexippus; H. sapiens: Homo sapiens; L. migratoria: Locusta migratoria; L. niger: Lasius niger; Mj: Marsupenaeus japonicus; M. musculus: Mus musculus; P. humanus corporis: Pediculus humanus corporis; P. monodon: Penaeus monodon; R. norvegicus: Rattus norvegicus; R. pedestris: Riptortus pedestris; S. mimosarum: Stegodyphus mimosarum; T. castaneum: Tribolium castaneum; Z. nevadensis: Zootermopsis nevadensis. (TIF) [file ppat.1006127.s010.tif]
